# Control of TGFβ signalling by ubiquitination independent function of E3 ubiquitin ligase TRIP12

**DOI:** 10.1038/s41419-023-06215-y

**Published:** 2023-10-20

**Authors:** Kripa S Keyan, Safa Salim, Swetha Gowda, Doua Abdelrahman, Syeda Sakina Amir, Zeyaul Islam, Claire Vargas, Maria Teresa Bengoechea-Alonso, Amira Alwa, Subrat Dahal, Prasanna R. Kolatkar, Sahar Da’as, Jerome Torrisani, Johan Ericsson, Farhan Mohammad, Omar M Khan

**Affiliations:** 1 College of Health and Life Sciences, Hamad Bin Khalifa University, Doha, Qatar; 2 Department of Research, Sidra Medicine, Doha, Qatar; 3 Qatar Biomedical Research Institute, Doha, Qatar; 4 Centre de Recherches en Cancérologie de Toulouse, Université de Toulouse, Inserm, CNRS, Université Toulouse III-Paul Sabatier, Toulouse, France

**Keywords:** Apoptosis, Molecular biology

## Abstract

Transforming growth factor β (TGFβ) pathway is a master regulator of cell proliferation, differentiation, and death. Deregulation of TGFβ signalling is well established in several human diseases including autoimmune disorders and cancer. Thus, understanding molecular pathways governing TGFβ signalling may help better understand the underlying causes of some of those conditions. Here, we show that a HECT domain E3 ubiquitin ligase TRIP12 controls TGFβ signalling in multiple models. Interestingly, TRIP12 control of TGFβ signalling is completely independent of its E3 ubiquitin ligase activity. Instead, TRIP12 recruits SMURF2 to SMAD4, which is most likely responsible for inhibitory monoubiquitination of SMAD4, since SMAD4 monoubiquitination and its interaction with SMURF2 were dramatically downregulated in *TRIP12*^*-/-*^ cells. Additionally, genetic inhibition of *TRIP12* in human and murine cells leads to robust activation of TGFβ signalling which was rescued by re-introducing wildtype TRIP12 or a catalytically inactive C1959A mutant. Importantly, TRIP12 control of TGFβ signalling is evolutionary conserved. Indeed, genetic inhibition of *Drosophila TRIP12* orthologue, *ctrip*, in gut leads to a reduced number of intestinal stem cells which was compensated by the increase in differentiated enteroendocrine cells. These effects were completely normalised in *Drosophila* strain where *ctrip* was co-inhibited together with *Drosophila SMAD4* orthologue, *Medea*. Similarly, in murine 3D intestinal organoids, CRISPR/Cas9 mediated genetic targeting of *Trip12* enhances TGFβ mediated proliferation arrest and cell death. Finally, CRISPR/Cas9 mediated genetic targeting of *TRIP12* in MDA-MB-231 breast cancer cells enhances the TGFβ induced migratory capacity of these cells which was rescued to the wildtype level by re-introducing wildtype TRIP12. Our work establishes TRIP12 as an evolutionary conserved modulator of TGFβ signalling in health and disease.

## Introduction

TGFβ family is a group of multifunctional cytokines involved in various cellular processes including embryogenesis, immune cell function, cell cycle regulation, tissue homoeostasis, extracellular matrix production, and tissue remodelling and repair [1–3]. TGFβ exerts its function via binding to and activating specific heteromeric serine-threonine activin receptor like kinases (ALK4/5) also known as type I and type II serine-threonine kinases [4]. The active TGFβ/Activin type I receptor directly phosphorylate the receptor-regulated SMADs (R-SMADs), SMAD2 and SMAD3 respectively. Phosphorylated R-SMADs interact with the common interacting partner and the major regulator of TGFβ/BMP signalling, SMAD4. The R-SMADs/SMAD4 heteromeric complex translocates to the nucleus leading to robust activation of downstream TGFβ pathway genes [5, 6].

In healthy tissues, TGFβ pathway activation leads to cell cycle arrest and apoptosis [7]. Due to its strong cytostatic ability, TGFβ is a potent tumour suppressor, and several members of TGFβ signalling pathway are frequently mutated in human cancers including colon and pancreatic cancer [8, 9]. Additionally, TGFβ may promote cancer growth by promoting epithelial-to-mesenchymal transition (EMT), cancer metastasis and invasion, and modulation of tumour microenvironment [10, 11]. Thus, TGFβ activity is tightly regulated by different mechanisms. For example, *SMAD7*, one of the downstream TGFβ pathway genes, contributes to the resolution of TGFβ activity by recruiting of E3 ubiquitin ligase SMURF2 to the TGFβR1 thereby facilitating its polyubiquitination and proteasome mediated degradation [12]. In addition, monoubiquitination of SMAD4 leads to its dissociation from the active R-SMADs complex and termination of TGFβ response [13]. Different E3 ubiquitin ligases including SMURF2 and TRIM33 have been implicated in inhibitory SMAD4 monoubiquitination [14–16].

The thyroid hormone receptor interactor protein 12 (TRIP12), also known as the E3 ubiquitin ligase for Arf, is a HECT-domain E3 ubiquitin ligase. TRIP12 is involved in DNA damage response, oncogenic stress, cell cycle control, and neurodegeneration [17–21]. Additionally, TRIP12 regulates response to PARP inhibitors in breast cancer cells [22] and we have shown that the genetic inhibition of *TRIP12* leads to the stabilisation of FBW7 protein, mitotic arrest, and enhanced cell death in response to Taxol treatment [23, 24]. Moreover, *Trip12* is essential for embryogenesis since *Trip12* mutant mice are not viable [25]. Interestingly, *TRIP12* variants are associated with intellectual disability and autism spectrum disorder [26, 27]. In addition, *TRIP12* mutations of unknown significance are reported in approximately 1–2% of human cancers [28, 29]. Despite the significance of TRIP12 in health and disease, the overall functional and cellular biology of TRIP12 is largely unknown.

Here, we show that TRIP12 interacted with SMAD4 and facilitated its inhibitory monoubiquitination by recruiting SMURF2. This interaction is necessary for efficient TGFβ mediated response. In the absence of *TRIP12*, SMURF2 recruitment to SMAD4 and its monoubiquitination is inhibited. This led to sustained activation of TGFβ pathway and upregulation of TGFβ mediated gene expression. These effects are seen in multiple mammalian cell lines including HEK293T, HepG2, and NIH3T3 fibroblasts. Interestingly, *trip12* deletion using CRISPR/Cas9 method in zebrafish embryos led to increased mortality and developmental defects in cr*Trip12* chimeras and genetic inhibition of *Drosophila* orthologue of *TRIP12* (*ctrip*) in intestinal stem cells (ISCs), led to a reduced number of ISCs and an increased number of Prospero positive differentiated enteroendocrine (EE) cells. The reduction in ISCs was rescued by simultaneous genetic inhibition of both *ctrip* and *Drosophila* orthologue of *SMAD4*, *Medea*. Finally, *Trip12* deletion enhanced TGFβ inhibitory effects in a mouse 3D intestinal organoid culture and, CRISPR/Cas9 mediated genetic inhibition of *TRIP12* in MDA-MB-231 cells enhanced TGFβ induced cell migration. Overall, our data suggest that regulation of TGFβ pathway by TRIP12 is evolutionarily conserved and understanding the mechanisms underlying this interaction can provide valuable insights into the intricate regulatory network of TGFβ signalling and its implications in various biological contexts.

## TRIP12 interacts with TGFβ/BMP pathway mediator SMAD4

**Fig. 1:**
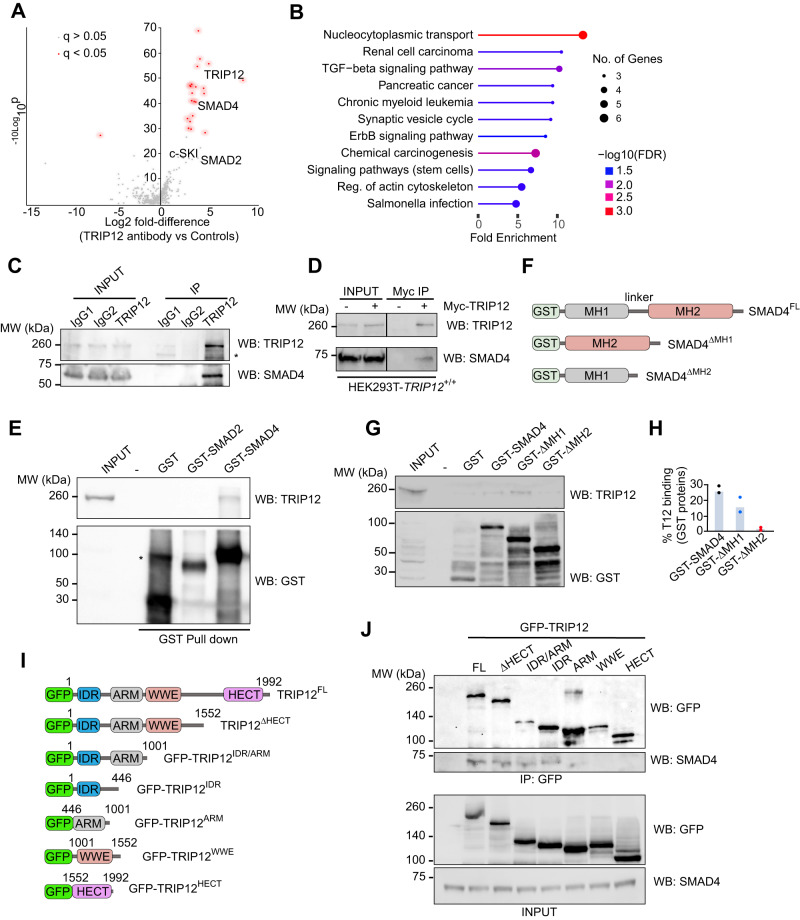
TRIP12 interacts with SMAD4 via its IDR and SMAD4’s MH2 domain. **A** Volcano plot showing the TRIP12 interactome. Significant interactions are denoted with red dots. **B** Gene ontology analysis of positive hits from experiment in (**A**), showing top enriched pathway proteins interacting TRIP12. **C** Immunoprecipitation (IP) validation of TRIP12/SMAD4 interaction using the same antibody used for proteomics experiment as in (**A**), followed by Western blots from HEK293T lysates. * represents a nonspecific band due to cross reactivity of anti-TRIP12 antibody. **D** IP**/**Western blot validation of Myc-TRIP12/SMAD4 interaction in HEK293T cell lysates using anti-Myc dynabeads. **E** Western blot validation of recombinant GST-SMAD4 pulls down endogenous TRIP12 in a GST pulldown experiment. GST only is used as a negative control. * represents a nonspecific band due to cross reactivity of anti-GST antibody. **F** Graphic demonstration of GST-tagged SMAD4 domains and SMAD4 deletion mutants. **G** Western blot validation of recombinant GST-SMAD4 and GST-ΔMH1 domain pull down endogenous TRIP12 in a GST pulldown experiment. GST only is used as a negative control. **H** Densitometric quantification of two independent GST pulldown experiments from experiments in (**F**). **I** Graphic demonstration of GFP-tagged TRIP12 wildtype and indicated deletion mutants. **J** IP**/**Western blot validation of GFP-TRIP12 and multiple GFP-TRIP12 deletion mutants’ interaction with endogenous SMAD4 in *TRIP12*^-/-^ HEK293T cells.

To understand the molecular pathways governed by TRIP12, we studied the TRIP12 interactome by performing TRIP12 immunoprecipitation using an antibody specific for endogenous TRIP12 followed by mass spectrometric (MS) analysis. We found that SMAD4—the major regulator of TGFβ/BMP signalling specifically interacted with TRIP12 (Fig. 1A). In addition, SMAD2 and c-SKI, two other major components of TGFβ signalling were also eluted with TRIP12 albeit at levels below our stringent criteria for TRIP12 specific interactors (Fig. 1A). Consistent with that, gene ontology (GO) analysis performed on TRIP12 interacting proteins also showed TGFβ pathway to be the topmost enriched pathway in our GO analysis (Fig. 1B). Further, we validated the MS data and confirmed that endogenous as well as a Myc-tagged TRIP12 both interacted with endogenous SMAD4 as confirmed by coimmunoprecipitation followed by Western blots (Fig. 1C, D). Conversely, GST-pulldown experiments confirmed direct interaction of GST-SMAD4 with endogenous TRIP12 while GST alone and GST-SMAD2 did not interact (Fig. 1E). SMAD4 interacted with TRIP12 via its MH2 domain, as SMAD4/TRIP12 interaction was significantly reduced when SMAD4 lacking MH2 domain (GST-ΔMH2) was used in pulldown experiments (Fig. 1F–H). Finally, to map SMAD4 interacting region on TRIP12, we generated several TRIP12 deletion mutants (Fig. 1I) and performed TRIP12/SMAD4 coimmunoprecipitations. Our data suggest that TRIP12 most likely interacted with SMAD4 via its internally disorganised region (IDR) as TRIP12 deletion mutants lacking IDR but not any other domains, failed to interact with endogenous SMAD4 (Fig. 1I, J). Thus, we demonstrate that TRIP12 interacts with SMAD4, and this interaction most likely occurs at the interface of SMAD4’s MH2 domain and TRIP12’s IDR region.

## TRIP12 is required for recruitment of E3 ubiquitin ligase SMURF2 to monoubiquitinate SMAD4

**Fig. 2:**
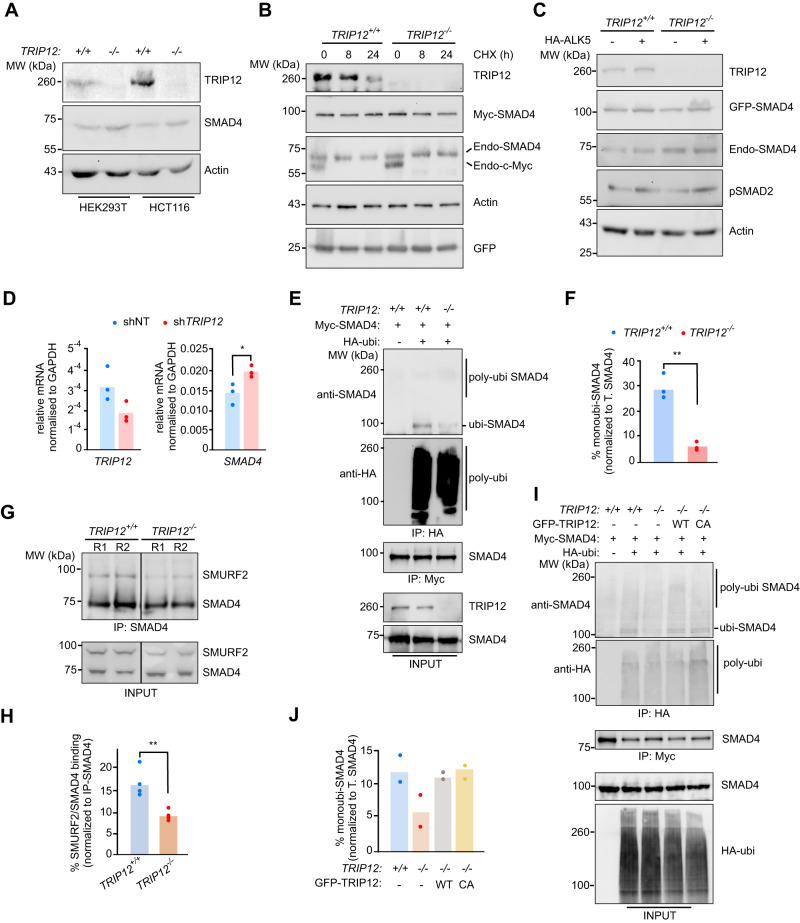
*TRIP12* inhibition does not affect SMAD4 protein stability but reduces its monoubiquitination. **A** Western blot comparison of SMAD4 protein levels in indicated *TRIP12*^+/+^ and *TRIP12*^-/-^ cell lines. **B** Western blots from HEK293T lysates showing indicated protein chase experiment. GFP is used as a loading control for transfection and Actin is used as an internal loading control. **C** Western blots showing indicated protein levels from *TRIP12*^+/+^ and *TRIP12*^-/-^ HEK293T cells ± HA-ALK5. Phospho-SMAD2 is used as a control for HA-ALK5 expression. **D** qRT-PCR for indicated genes in HepG2 cells stably expressing sh-non template (*NT*) or sh*TRIP12*. **E** Western blots showing indicated proteins after denaturing IP with HA-beads (ubiquitinated SMAD4) and Myc-beads (Control/SMAD4) from HEK293T cells of indicated genotypes. **F** Densitometric quantification from three independent experiment as in (**E**). Statistics were done by student’s *t* test. **G** Western blots showing SMURF2/SMAD4 interaction followed by SMAD4 IP in HEK293T cells of indicated genotypes. R1 and R2 are two different replicates in the same experiment. **H** Densitometric quantification from four independent experiments from (**G**). The dots represent a single SMURF2 densitometric value normalised to immunoprecipitated SMAD4 in the same experiment. **I** Western blots showing indicated proteins after denaturing IP with HA-beads (ubiquitinated SMAD4) and Myc-beads (Control/SMAD4) from HEK293T cells of indicated genotypes. **J** Densitometric quantification from two independent experiments as in (**I**). Statistics were done by student’s *t* test in (**D,** **H**, and **F**) and each dot in the plots represent mean of triplicates from three independent experiments unless otherwise stated. *P* = * < 0.05 and ** < 0.01.

To functionally characterise the epistasis between TRIP12 and SMAD4, we investigated the endogenous levels of SMAD4 protein in *TRIP12*^*-/-*^ cell lines compared to the wildtype controls. We noticed a subtle increase in SMAD4 protein levels in both HEK293T and HCT116 *TRIP12*^*-/-*^ cell lines (Fig. 2A). To check if TRIP12 regulates SMAD4 protein stability, we performed cycloheximide protein chase assays. Surprisingly, SMAD4 protein stability was largely unaffected in response to cycloheximide regardless of *TRIP12* status (Fig. 2B), whereas TRIP12 itself and an additional control c-MYC, showed robust protein degradation over time in cycloheximide treated cells albeit at different time kinetics as expected for two largely distinct class of proteins (Fig. 2B). Additionally, Myc-SMAD4 protein stability was neither affected by treatment with a proteasomal inhibitor (MG132) nor by co-overexpression of TRIP12 wildtype, ΔHECT, ARM, and HECT domains (Supplementary Fig. 1A). Next, we looked at the possibility that TRIP12 might regulate SMAD4 protein levels in response to TGFβ signalling. For this, we co-overexpressed a constitutively active ALK5 (TGFβRI) mutant and GFP-SMAD4 and performed Western blots to check the protein levels of both endogenous as well as GFP-SMAD4 proteins. We reasoned that, if TRIP12 controls SMAD4 protein stability, both endogenous and GFP-tagged, SMAD4 proteins must be altered in *TRIP12*^*-/-*^ cells. However, if *TRIP12* deletion causes increase in SMAD4 protein due to transcriptional or posttranscriptional changes then only endogenous protein should be altered. GFP-SMAD4 was largely unaffected in *TRIP12*^-/-^ knockout cells and ALK5 overexpression had little to no effect on its levels (Fig. 2C). We confirmed ALK5 activity with pSMAD2 and found a strong induction of pSMAD2 in HA-ALK5 overexpressing samples (Fig. 2C). However, we found consistent increase in endogenous SMAD4 levels in the absence of *TRIP12* which was also unaffected by ALK5 overexpression in *TRIP12*^*-/-*^ cells (Fig. 2C). Interestingly, *SMAD4* mRNA was significantly increased in *TRIP12* knockdown cells suggesting that the increase in SMAD4 protein in the absence of *TRIP12* is most likely a transcriptional event (Fig. 2D), that is, increased *SMAD4* mRNA most likely leads to the subtle increase in SMAD4 protein levels in *TRIP12*^-/-^ cells and TRIP12 does not regulate SMAD4 protein stability.

Monoubiquitination of SMAD proteins including SMAD4 is required to control their cellular localisation, protein-protein interactions, and TGFβ signalling [30, 31]. Largely, monoubiquitination inhibits association of SMAD4 with R-SMADs to prevent formation of a stable transcriptional complex and inhibit TGFβ signalling. Several E3 ubiquitin ligases including β-TRCP1, SMURF2, and TRIM33 are known to ubiquitinate SMAD4 [31], with E3 ligase SMURF2 proposed to only monoubiquitinate SMAD4 to restrain TGFβ pathway activation [32]. *TRIP12* depletion near completely blocked monoubiquitination of SMAD4 protein (Fig. 2E, F). In these experiments we detected low levels of SMAD4 polyubiquitylation as well which was unaffected between the wildtype and *TRIP12*^-/-^ cells. Thus, we hypothesised that TRIP12 regulated SMAD4 monoubiquination must be mediated by SMURF2. To test that, we performed co-immunoprecipitation of SMURF2 and SMAD4 and found that the SMURF2 recruitment on SMAD4 was significantly downregulated in *TRIP12*^*-/-*^ cells (Fig. 2G, H). Strikingly, not only SMAD4 monoubiquitination but also SMURF2 interaction with SMAD4 was restored by re-introducing wildtype as well as a catalytically dead TRIP12-C1959A mutant in *TRIP12* knockout cells (Fig. 2I, J and Supplementary Fig. 1B).

To this end, our data suggest a model where TRIP12 recruits SMURF2 to carry out SMAD4 ubiquitination. But it is possible that TRIP12 may directly ubiquitinate SMAD4 and even the C1959A mutant possess ubiquitin ligase activity sufficient to monoubiquitinate SMAD4. Thus, to overrule this possibility, we first validated the plasmid sequences of TRIP12 wildtype and C1959A mutant via sanger sequencing and confirmed that Cysteine 1959 is indeed mutated to Alanine in this construct (Supplementary Fig. 1C). Second, to exclude the possibility that a nearby ‘Cys’ or any other residue in TRIP12 HECT domain might compensate for ubiquitin ligase activity in TRIP12 C1959A mutant, we purified wildtype and C1959A HECT domains (Supplementary Fig. 1D) and performed in vitro ubiquitination assay. Indeed, wildtype TRIP12 efficiently ubiquitinated free ubiquitin in collaboration with two different E2 enzymes whereas C1959A mutant largely lacked this activity (Supplementary Fig. 1E). In agreement with that, TRIP12 HECT domain failed to directly ubiquitinate SMAD4 in an in vitro ubiquitination reaction carried out in presence of multiple combinations of UBE1/UBE2 with TRIP12-HECT domain (Supplementary Fig. 1F). Thus, the E3 ubiquitin ligase activity of TRIP12 is not required for SMAD4 monoubiquitination rather TRIP12 recruits SMURF2 to carry out SMAD4 monoubiquitination.

## TRIP12 controls TGFβ transcriptional activity via SMAD4

**Fig. 3:**
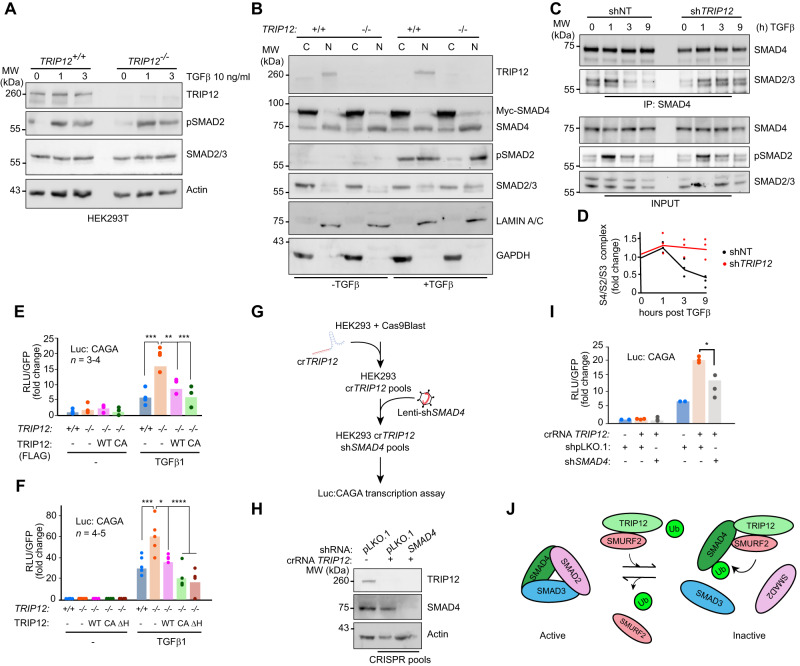
*TRIP12* inhibition increases TGFβ induced luciferase transcriptional response via SMAD4. **A** Western blots comparison of SMAD2/3 protein phosphorylation status in *TRIP12*^+/+^ and *TRIP12*^-/-^ HEK293T cells. **B** Western blots from sub-cellular fractions (C = cytoplasmic, and N = nuclear) of *TRIP12*^+/+^ and *TRIP12*^-/-^ HEK293T cells showing indicated protein levels. LAMIN A/C and GAPDH are used as nuclear and cytoplasmic loading controls respectively. **C** Western blot showing IP SMAD4/R-SMAD complex formation in stable HepG2 cells expressing indicated shRNA, Phospho-SMAD2 is used as a control for TGFβ pathway activation. **D** Densitometric quantification from 3 independent experiments as in (**C**). Each dot represents a single densitometric value from experiments in (**C**). **E** Bar graphs showing Luc:CAGA TGFβ transcriptional activity as relative luciferase units (RLU) in *TRIP12*^+/+^ and *TRIP12*^-/-^ HEK293T cells ectopically expressing indicated plasmids. **F** Bar graphs showing Luc:CAGA TGFβ transcriptional activity as RLU in *TRIP12*^+/+^ and *TRIP12*^-/-^ HEK293T cells ectopically expressing indicated plasmids. **G** Schematic for generation of *TRIP12*/*SMAD4* double knockdown cells. **H** Western blots validation for indicated proteins from HEK293T cells of indicated genotypes and stable shRNA expression from (**G**). **I** Bar graphs showing Luc:CAGA TGFβ transcriptional activity in cells stably expressing indicated plasmids from (**G**). Statistics were done by student’s *t* test, *p* = * < 0.05. **J** Scheme of proposed mechanism of TGFβ regulation by TRIP12. Statistics were done by Two-way ANOVA in (**E**) and (**F**), *p* = * < 0.05, ** < 0.01, *** < 0.001, & **** < 0.0001 and shown for only statistically significant comparisons. Each dot in the plots represent mean of duplets from *n* = 3–5 independent experiments unless otherwise stated.

Having established the epistasis between TRIP12 and SMAD4, we set to understand the function of TRIP12 in TGFβ signalling. TGFβ treatment leads to robust phosphorylation of SMAD2 (p-SMAD2) but *TRIP12* deletion does not affect the levels and duration of p-SMAD2 in two different cell lines (Fig. 3A and Supplementary Fig. 2A). Next, we performed cytoplasmic and nuclear subcellular fractionation and found that both exogenous and endogenous SMAD4 protein levels were higher in nuclear extracts of *TRIP12*^*-/-*^ cells compared to wildtype controls regardless of TGFβ treatment (Fig. 3B), which could be a result of reduced SMAD4 monoqubiquitination as previously suggested [32]. Although cytoplasmic/nuclear SMAD4 protein shuttling was unaffected by TGFβ treatment, the total- and p-SMAD2 levels in nuclear extracts of *TRIP12*^*-/-*^ cells were higher compared to the wildtype controls (Fig. 3B). The nuclear accumulation of p-SMAD2 in *TRIP12*^*-/-*^ cells most likely led to sustained activation of SMAD2/3/4 complex in *TRIP12* knockdown HepG2 cells (Fig. 3C, D). Consistent with this result, a TGFβ responsive synthetic 12XCAGA promoter luciferase reporter (Luc:CAGA) assay showed more than two-fold transcriptional activity in *TRIP12*^*-/-*^ cells in response to TGFβ, which was normalised to wildtype levels by transient overexpression of human wildtype- as well as C1959A mutant TRIP12 (Fig. 3E), and endogenous overexpression of *TRIP12* using CRISPR/Cas9-guided synergistic activation mediator from our previously published HEK293T cells [23] completely blocked Luc:CAGA TGFβ reporter activity (Supplementary Fig. 2B, C). Similar results were obtained with another TGFβ responsive Luc:PAI reporter assay (Supplementary Fig. 2D). Strikingly, a HECT domain deleted TRIP12 mutant (TRIP12-ΔHECT) was sufficient to block TGFβ reporter Luc:CAGA activity (Fig. 3F). Enhanced Luc:CAGA activity was also observed in TGFβ responsive- human HepG2 cells, and murine NIH3T3 fibroblasts expressing lentivirus mediated sh*TRIP12* and sh*Trip12* respectively (Supplementary Fig. 2E–G). Additionally, TGFβ3 from a different vendor also induced higher Luc:CAGA promotor activity in *TRIP12*^*-/-*^ cells, confirming that the increase in TGFβ signalling in *TRIP12*^*-/-*^ cells is not restricted only to TGFβ1 (Supplementary Fig. 2H). Finally, Luc:CAGA transcriptional activity was significantly reduced in cells with co-inhibition of *TRIP12* and *SMAD4* (Fig. 3G–I), confirming the requirement of SMAD4 in enhanced TGFβ signalling in *TRIP12*^*-/-*^ cells. These results indicate that TRIP12 controls TGFβ activity independent of its E3 ubiquitin ligase activity and via negative regulation of SMAD4 by recruiting E3 ligase SMURF2, and the deletion of *TRIP12* leads to sustained activation of TGFβ signalling (Fig. 3J).

## *TRIP12* depletion leads to enhanced TGFβ target gene expression and developmental defects in a zebrafish model

**Fig. 4:**
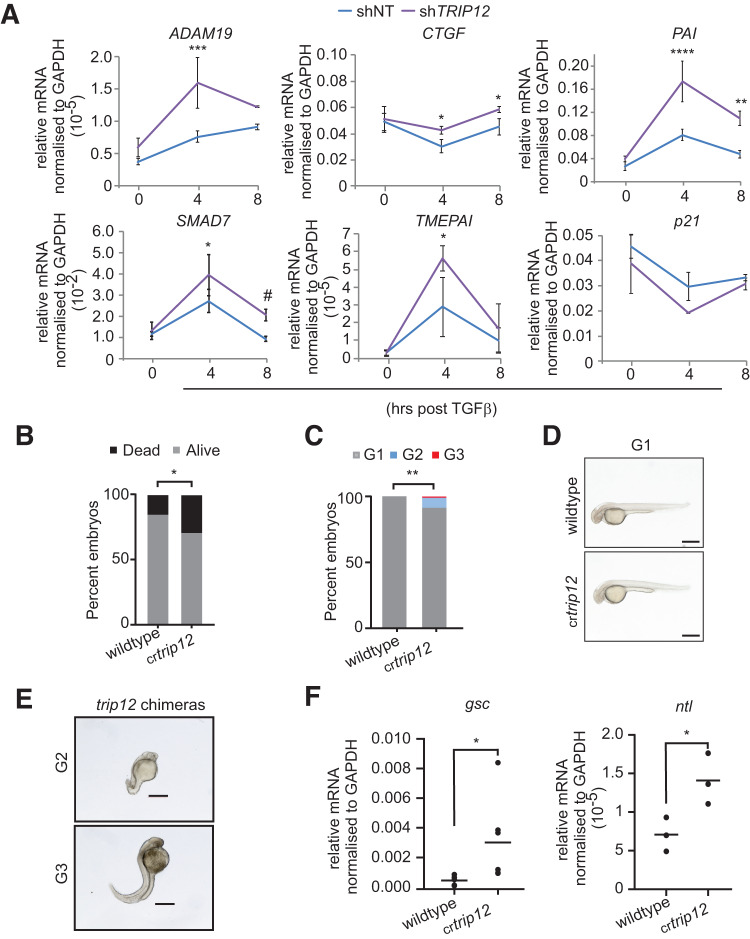
*TRIP12* controls endogenous TGFβ pathway gene regulation. **A** qRT-PCR data of six selected TGFβ target genes in HepG2 cells stably expressing shNT and sh*TRIP12*, stimulated with 10 ng/ml TGFβ for indicated time intervals. Statistics are done by Two-way ANOVA from at least three independent experiments, bars are standard deviation, *p* = * < 0.05, *** < 0.01, **** < 0.001. **B** Zebrafish embryo survival data. **C** Bar graphs showing the distribution of zebrafish embryos according to their respective developmental stages where G1 = severe abnormality G2 = mild abnormality, and G3 = normal. **D**, **E** Representative images of zebrafish embryos with G1, G2, and G3 phenotypes from (**C**). Statistics are done by Fisher’s exact *t* test, *p* = *< 0.05 and ** < 0.01. **F** qRT-PCR analysis of two well established TGFβ/BMP pathway genes from zebrafish embryos 48–96 days post co-injection of CRISPR/Cas9 with control (wildtype) or chemically synthesized CRISPR gRNA targeting *ctrip12* (cr*trip12*). Statistics were done by student’s *t* test and each dot in the plots represent a mean value from three replicates in *n* = 3–5 pools of zebrafish embryos*, p* = * < 0.05.

To this end, we established that TRIP12 controls TGFβ signalling via its ubiquitination independent function. To confirm if TRIP12 regulates endogenous TGFβ target gene expression, we checked the levels of six well characterised TGFβ targets in control and *TRIP12*-knockdown cells in response to TGFβ [33]. Consistent with sustained activation of TGFβ signalling in *TRIP12* depleted cells, four out of six TGFβ target genes *ADAM19, CTGF, PAI*, and *SMAD7* showed significant and sustained upregulation in *TRIP12-*knockdown cells (Fig. 4A). *TMEPAI* mean expression was significantly increased at 4-h timepoint, while *P21* gene expression levels were unaffected by *TRIP12* knockdown (Fig. 4A). Similar result of enhanced TGFβ target gene expression was obtained in NIH3T3 sh*Trip12* mouse fibroblasts compared to the controls (Supplementary Fig. 3A). These results strongly suggest that TRIP12 regulate TGFβ signalling in both human and murine cells.

To test if TRIP12 controls TGFβ/BMP gene expression in vivo, we used a zebrafish model to study TGFβ signalling as TGFβ/BMP signalling is required for the early development of vertebrates [34]. While genetic inhibition of *Trip12* is embryonic lethal in mice [25] and *Drosophila* (our unpublished data), its effect on zebrafish development is not known. Thus, to study the impact of *TRIP12* deletion in development, we employed the zebrafish model and targeted *TRIP12* orthologue of zebrafish, *trip12* (ENSDARG00000061397, ENSG00000153827), by CRISPR/Cas9 system and generated zebrafish *trip12* crispants (Supplementary Fig. 3D, E). The zebrafish mortality rate increased in *trip12* crispants; additionally, only *trip12* crispants (~20%) but not control showed developmental defects (Fig. 4B–E). The developmental defects of *trip12* crispants were most likely due to deregulated TGFβ signalling given that two well-established TGFβ target genes *gsc* and *ntl1* were significantly upregulated in *trip12* crispants group (Fig. 4F). Thus, TRIP12 controls TGFβ signalling both in mammalian cells as well as in a zebrafish model.

## *ctrip* controls tissue homoeostasis in *Drosophila* gut via negative regulation of TGFβ signalling

**Fig. 5:**
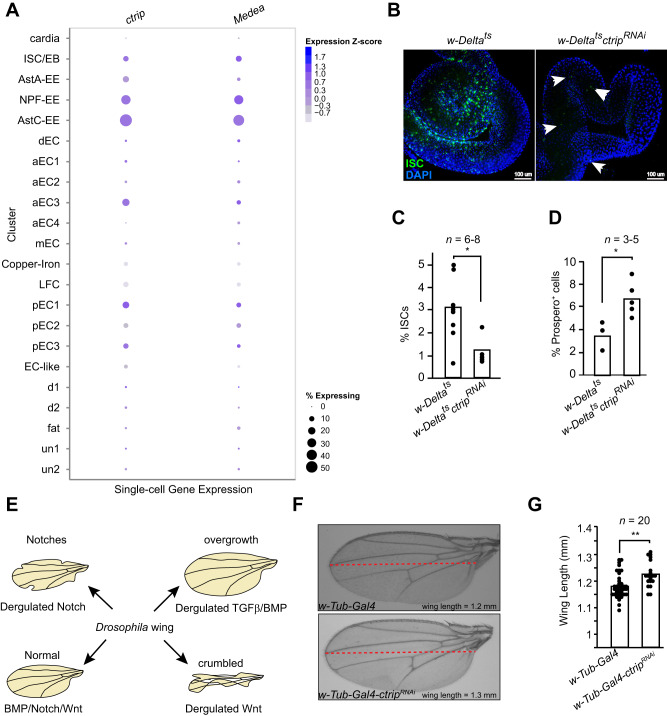
*Drosophila* Trip12 regulates gut homoeostasis via control of TGFβ signalling in vivo. **A** Single cell RNA-sequencing data obtained from https://www.flyrnai.org/scRNA/gut/ showing strong pattern of *ctrip* and *Media* co-expression in *Drosophila* intestinal cells including intestinal stem cells/erythroblasts (ISCs/EBs), enteroendocrine cells including Allostatins A/C (Ast) and neuropeptide F (NPF) positive (EEs), anterior enterocytes (aECs), large flat cells (LFC), and posterior ECs (pECs). **B** Immunofluorescence images of control and *ctrip* targeted *Drosophila* gut with ISCs marked by GFP expression. **C** Flow cytometry confirmation of reduced ISCs in *ctrip* targeted *Drosophila* gut. **D** Quantification of Prospero+ differentiated cells in the guts from the indicated genotypes. **E** Schematic description of *Drosophila* wing development by major signalling pathways conserved between *Drosophila* and mammals. **F** Representative images of *Drosophila* wings from indicated genotypes. **G** Quantification of *Drosophila* wing length from indicated genotypes. Statistics were done by student’s *t* test. Each dot in the plots represent a mean value from 2–3 replicates in *n* = 3–8 independent pools of indicated genotypes in (**C**) and (**D**) and in *n* = 20 animals in (**G**). *p* = * < 0.01 and ** < 0.001.

*Drosophila* is a useful model to study adult gut tissue homoeostasis [35]. The pathways that control ISCs renewal and differentiation are relatively conserved between *Drosophila* and mammals [36]. Having established the requirement of TRIP12 in TGFβ signalling in vitro and in vivo we set to test the cellular biology of TRIP12 in the context of adult tissue homoeostasis in *Drosophila*. First, we explored the gene expression pattern of *ctrip* and *Medea* genes, the *Drosophila* orthologues of human *TRIP12* and *SMAD4* respectively. For this, we used a previously published singe-cell RNA sequencing dataset of *Drosophila* intestine (https://www.flyrnai.org/scRNA/gut/) [37]. We found a strikingly similar pattern of co-expression of both genes from the highest to the lowest expression in the order of EE, ISCs and enteroblasts (EB), and enterocytes (EC) (Fig. 5A). Second, to understand the functional biology of TRIP12, we used a temperature sensitive *Gal4*-UAS based RNAi system which allowed simultaneous expression of *ctrip* RNAi and labelling of ISCs and EBs with GFP [38]. Interestingly, *ctrip* knockdown in the ISCs of adult fly led to a sharp reduction in number of ISCs/EBs compared to controls as judged by immunofluorescence microscopy and flow cytometric analyses (Fig. 5B, C). Conversely, *ctrip* inhibition increased the number of Prospero positive EE cells compared to the controls suggesting a possibility that enhanced differentiation of ISCs/EBs rather than a proliferation defect is responsible for the reduction of ISCs/EBs in *ctrip* inhibited *Drosophila* gut (Fig. 5D).

To understand the molecular pathways governed by *ctrip* in *Drosophila* cell fate decisions, we used the *Drosophila* wing as a model. The development and growth of *Drosophila* wing is maintained by an intricate balance between several well conserved pathways including the WNT, Notch, & TGFβ/BMP signalling (Fig. 5E) [39–41]. Interestingly, wing specific *ctrip* knockdown using *Tub-Gal4* based RNAi in *Drosophila* led to increase in *Drosophila* wing length as judged by quantification of wing length (Fig. 5F, G). The absence of a classical WNT or Notch phenotype in *ctrip* knockdown *Drosophila* wing suggests a possibility that *ctrip* controls wing growth either by TGFβ/BMP pathway or via an unknown mechanism.

**Fig. 6:**
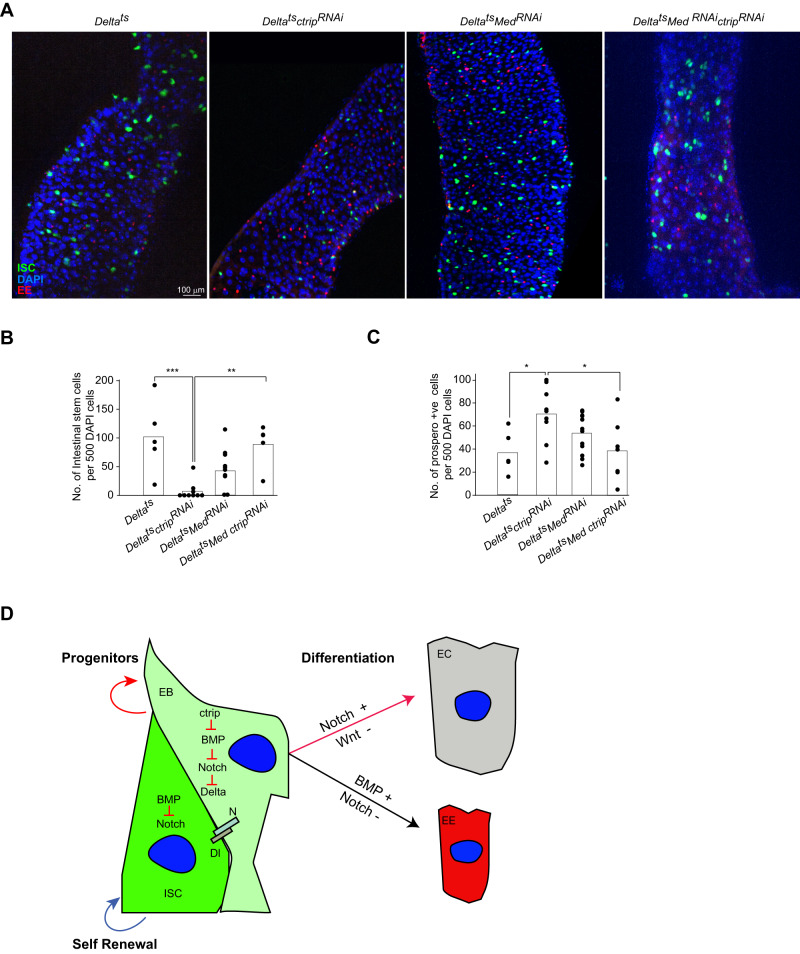
Inhibition of *Drosophila* SMAD4 orthologue *Medea* restores ISCs number in *ctrip* deficient *Drosophila* gut. **A** Immunofluorescence images of *Drosophila* gut showing ISCs marked by GFP expression and Prospero + EEs by IF, from the indicated genotypes. Quantification of ISCs (**B**)*,* and EEs (**C**)*,* from experiment in (**A**). **D** Graphical abstract of ISCs renewal and differentiation by multiple signalling pathways highly conserved between *Drosophila* and mammals. Statistics were done by One-way ANOVA. *p* = * < 0.05, ** < 0.01, and *** < 0.001. Each dot in the plots represents mean cell counts of individual pools of guts from respective genotypes.

To test this hypothesis, we generated *Drosophila* lines in which we either inhibited *ctrip* alone or in combination with *Medea*. Strikingly, *ctrip/Medea* double RNAi-based knockdown restored the reduced number of ISCs/EBs in *Drosophila* and reversed the differentiated cell phenotype back to the control levels (Fig. 6A–C). In *Drosophila* gut, high TGFβ/BMP signalling maintains low Notch levels and this arm of signalling favours differentiation of progenitors to EE [42, 43]. Our data suggest that *ctrip* in *Drosophila* ISCs and EBs maintain a threshold of BMP signalling just about enough to establish gut homoeostasis. In the absence of *ctrip*, this balance tilts towards higher differentiation of ISCs and EBs to EE lineage (Fig. 6D). These results suggest that *ctrip* controls TGFβ/BMP signalling in vivo, and this is required for the maintenance of *Drosophila* gut biology.

## *Trip12* inhibition potentiates TGFβ induced proliferation arrest and apoptosis in mouse intestinal organoids

**Fig. 7:**
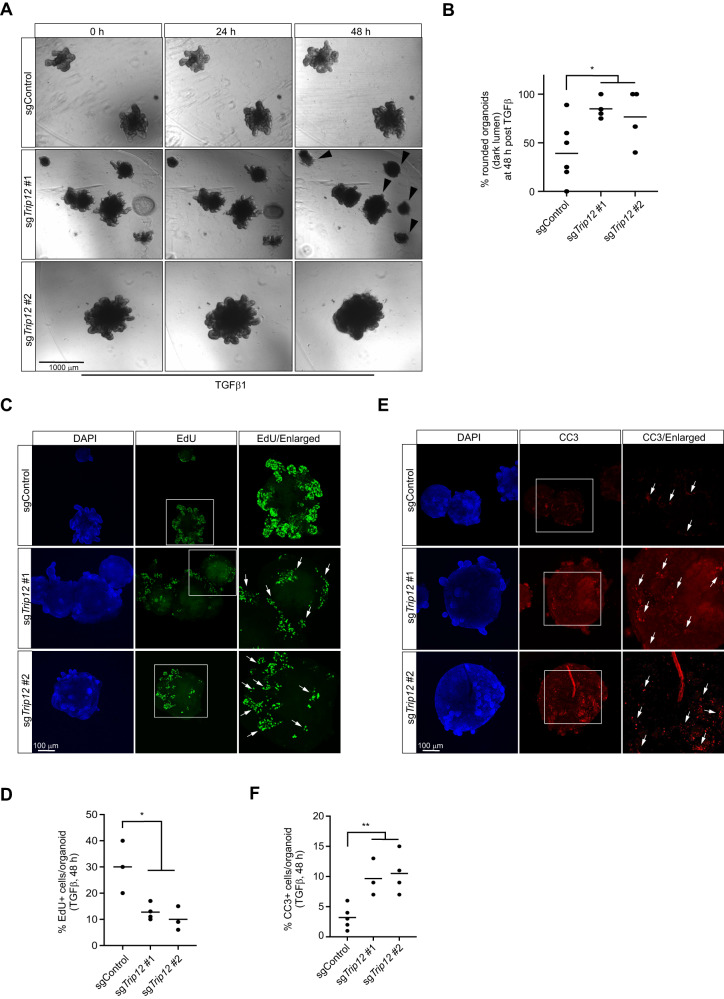
*Trip12* deletion potentiates TGFβ response in primary mouse intestinal organoids. **A** Bright field images of mouse intestinal organoids treated with 1 ng/ml TGFβ for indicated time intervals. **B** Quantification of apoptotic looking regressing organoids from experiment in (**A**). **C** Immunofluorescence images of mouse intestinal organoids treated with 1 ng/ml TGFβ for 48 h. Proliferating ISCs and TA are labelled with EdU and stained using Click-iT^ΤΜ^ Alexa Fluor kit. Arrows point at regressing budding structures that lose EdU incorporation compared to controls. Nuclei are counterstained with DAPI. **D** Quantification of EdU positive cells in organoids from (**C**). EdU positive cells were quantified from three independent images taken at different levels on at least 3–5 different organoids from two independent experiments. **E** Immunofluorescence for CC3 positive apoptotic cells in organoids from indicated genotypes. Nuclei are counterstained with DAPI. **F** CC3 positive cells are quantified from three independent images taken at different levels on at least 3–5 different organoids from two independent experiments and presented as % of DAPI positive cells. Statistics were done by One-way ANOVA. Each dot in the plots represents a mean value of cell count from up to 3 independent images from two different experiments, *p* = * < 0.05 and ** < 0.01.

To check if TRIP12 also mediates TGFβ activities in mammalian intestinal cells, we used CRISPR/Cas9 based genetic targeting of mouse *Trip12* gene in intestinal organoids and studied their cellular biology. We used two independent sgRNA to target *Trip12* locus and confirmed the genetic manipulation of *Trip12* gene by a T7 restriction endonuclease method (Supplementary Fig. 4A, B). Interestingly, incubation of low dose TGFβ which is well tolerated by the control organoids, led to dramatic regression of sg*Trip12* organoids by 48 h and they appeared as apoptotic looking circular bodies with dark black central core typical of lumen full of dead cells (Fig. 7A, B). At higher dose of 10 ng/ml TGFβ, all organoids regardless of *Trip12* status regressed to appear like apoptotic bodies (Supplementary Fig. 4C). To test if the change in morphology of sg*Trip12* organoids is indeed due to the inhibition of proliferating ISCs, we pulsed the organoid culture with EdU for 2 h and performed the Click-IT chemistry assay followed by immunofluorescence microscopy. As expected, TGFβ treatment led to robust reduction of EdU incorporation in sg*Trip12* organoids compared to the controls (Fig. 7C, D). Consistent with this observation, the number of cleaved caspase-3 (CC3) positive cells was also increased in sg*Trip12* organoids suggestive of increased apoptosis in those organoids in response to low dose TGFβ (Fig. 7E, F). Thus, our data suggest that TRIP12 is an essential mediator of TGFβ signalling in *Drosophila* as well as murine intestinal cells.

## TRIP12 inhibits cancer cell migration

**Fig. 8:**
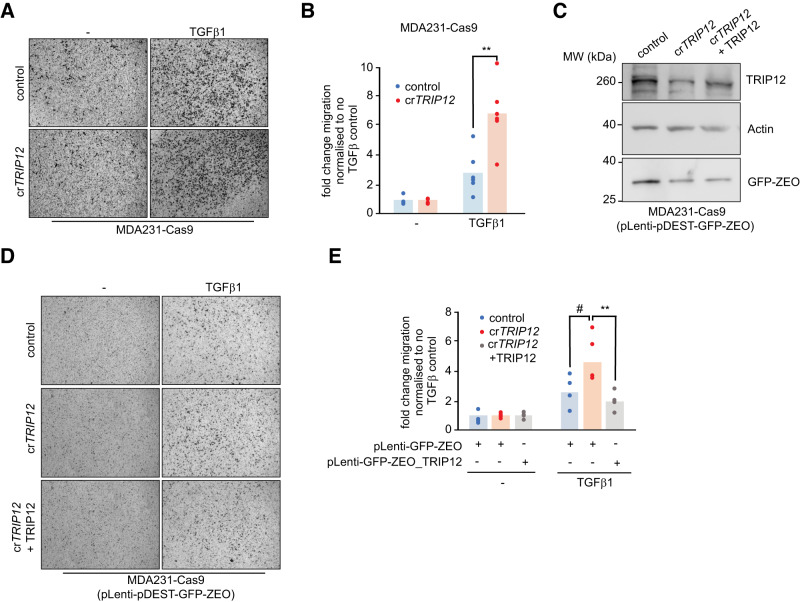
TRIP12 controls metastasis of MDA-MB-231 cells. **A** Bright field images of migrated MDA-MB-231 cells ± TGFβ treatment from indicated genotypes. **B** Quantification of migrated cells from 5 independent experiments in (**A**). **C** Western blot confirmation of TRIP12 overexpression in MDA-MB-231 cells from indicated genotypes. **D** Bright field images of migrated MDA-MB-231 cells ± TGFβ treatment from indicated genotypes. **E** Quantification of migrated cells from 4 independent experiments in (**D**). Statistics were done by One-way ANOVA. Each dot in the plots represents a mean value of cell count from 4–5 random images of migrated cells from each insert, *p* = * < 0.05, ** < 0.01, and # < 0.07.

To this end, we established TRIP12 as a modulator of TGFβ signalling in normal healthy tissues. To check if TRIP12 can potentially regulate TGFβ mediated cancer cell migration, we generated CRISPR/Cas9 targeted cr*TRIP12* pools of MDA-MB-231 cells (Supplementary Fig. 5). When stimulated with TGFβ in a transwell chamber assay, cr*TRIP12* cells migrated approximately 2–3-fold higher than their wildtype counterparts (Fig. 8A, B). Furthermore, reintroducing wildtype TRIP12 in cr*TRIP12* MDA-MB-231 cells, normalised the increased migration of those cells to the wildtype levels (Fig. 8C–E). Thus, TRIP12 regulates TGFβ signalling in normal as well as cancer cells.

## Discussion

TGFβ pathway is the master regulator of cell proliferation and death. Deregulation TGFβ signalling contributes to developmental disorders and cancer. Understanding the molecular mechanisms of TGFβ pathway deregulation may help in understanding the underlying causes of some of these conditions. Previous work has extensively characterised the regulation of TGFβ signalling by posttranslational modulations of members of TGFβ signalling pathway [44–46]. Here, we made an unexpected discovery that E3 ubiquitin ligase TRIP12 which was previously shown to be required in diverse cellular processes including DNA damage, cell cycle regulation, chemotherapy resistance, and neurodegeneration [18, 19, 22, 24], controls TGFβ pathway activity independent of its E3 ubiquitin ligase activity.

Our data show that TRIP12 interacts with SMAD4 via its IDR region. The IDR region may confer DNA binding ability to proteins. TRIP12 IDR has been shown to bind directly to DNA elements [47]. Given the fact that TRIP12 is involved in DNA damage response and gene expression, it is plausible that the IDR domain of TRIP12 is its DNA binding domain. Interestingly, we show that SMAD4 interacts with TRIP12 IDR via its MH2 domain. This interaction is not dependent on other protein-protein interaction domains including WWE and ARM domain in TRIP12. Thus, TRIP12 may use its different domains to selectively interact with its broad range of substrates. The presence of low levels of other TGFβ pathway proteins including SMAD2 and c-SKI in our IP-MS experiment suggests a possibility that TRIP12 via its IDR domain may recruit other components of TGFβ pathway to SMAD4 (Fig. 1), but this possibility needs to be further validated.

Previous work have suggested that SMAD4 is subjected to proteasomal degradation and several E3 ubiquitin ligases have been linked with SMAD4 polyubiquitination [15, 48]. Interestingly our data is contrary to some of those findings. First, we do not find any change in SMAD4 protein levels in the presence of a commonly used proteasomal inhibitor, MG132. Second, SMAD4 protein stability over time as judged by cycloheximide treatment followed by Western blots, show minimal change in SMAD4 protein levels even after 24 h, suggesting that SMAD4 is relatively a stable protein. Finally, both endogenous as well as ectopically expressed SMAD4 protein levels were unaffected by MG132 or cycloheximide treatment regardless of the duration of treatment overruling the possibility of any transcriptional changes due to these treatments. These contrasting results may reflect the cell type specific and context dependent regulation of SMAD4 protein. Indeed, previous work that linked SMAD4 polyubiquitination with its proteasomal degradation was largely done in cancer cell lines including with use of some cancer associated *SMAD4* mutants [33, 49–51] (Fig. 2 and Supplementary Fig. 1).

Surprisingly, endogenous SMAD4 is largely localised to the nucleus even in the absence of TGFβ incubation while SMAD2 readily shuttles to the nucleus only upon TGFβ stimulation, and its phosphorylated form is retained longer in the nucleus in the absence of *TRIP12*, which may explain sustained activation of SMAD4/SMAD2 complex in those cells (Fig. 3). The SMAD4 nuclear localisation in the absence of TGFβ stimulation might be due to underlying active BMP signalling or via another pathway which is independent of TGFβ/BMP signalling. The SMAD4 monoubiquitination results in its dissociation from the R-SMADs and resolution of TGFβ response [52]. We show that SMAD4 monoubiquitination is dramatically inhibited in *TRIP12*-knockout cells. In our subsequent biochemical analysis, we do not find any direct ubiquitination of SMAD4 by recombinant TRIP12 HECT domain. Instead, we found reduced SMURF2 recruitment to SMAD4 in *TRIP12*-knockout cells which was restored back to the wildtype levels when ectopically expressed GFP-tagged TRIP12 wildtype as well as a C1959A mutant is introduced in these cells (Supplementary Fig. 1B). Consistent with that, both TRIP12 wildtype and C1959A mutant restored the monoubiquitination of SMAD4 in *TRIP12*-knockout cells. These results strongly suggest that the monoubiquitination of SMAD4 is largely dependent on TRIP12’s ability to recruit SMURF2 to SMAD4 (Fig. 2G–J).

Genetic inhibition via shRNA or CRISPR/Cas9 mediated deletion of *TRIP12* gene results in robust activation of TGFβ signalling (Fig. 3D, E). This increased signalling is completely blocked by reintroducing wildtype, C1959A, and ΔHECT TRIP12 mutants (Fig. 3F). TRIP12 contains several defined and undefined domains including the IDR, WWE, ARM, and the HECT domain. Previous work have established the notion that the E3 ubiquitin ligase activity of TRIP12 drives its molecular functions. Interestingly, TRIP12 may also regulate cell cycle dynamics independent of its E3 ligase activity [18], and a recent work identified ubiquitination independent function of another E3 ubiquitin ligase HERC3 in the regulation of transcriptional co-activators YAP/TAZ’s activity [53]. Along the same lines, we show that TRIP12 can also regulate TGFβ activity independent of its E3 ubiquitin ligase activity and our data suggest that TRIP12 biological functions may extend beyond its catalytic activity.

Our work potentially establishes the role of TRIP12 for the first time in the regulation of TGFβ biology in multiple models. For example, we show that *TRIP12* deletion in mammalian cell lines, zebrafish, and *Drosophila* leads to phenotypes highly concordant with deregulated TGFβ signalling. First, we show that in several human cell lines and mouse NIH3T3 fibroblasts the levels and duration of TGFβ induced response and target genes are increased (Fig. 4A and Supplementary Fig. 4A). Second, two well established TGFβ target genes were upregulated in *trip12* crispants zebrafish embryos during development (Fig. 4F). This increase in TGFβ signalling in zebrafish embryos may also contribute to developmental defects in these embryos (Fig. 4B–E) [34]. However, we do not exclude the possibility that zebrafish developmental defects may arise from a potential deregulation of other pathways previously attributed to *Trip12* deficiency in mice [25]. Third, in *Drosophila* gut we find a sharp reduction of ISCs which was compensated by the increase in differentiated EE cells. Indeed, the numbers of ISCs and EEs were restored to control levels in *Drosophila* when *ctrip* and *Medea* genes were simultaneously inhibited, suggesting that the ISCs renewal and differentiation phenotype in this model is largely driven by TGFβ/BMP signalling (Fig. 6). The reduction of ISCs in *ctrip* inhibited *Drosophila* gut may also arise from increased cell death of ISCs, however, we do not find any evidence of enhanced ISCs cell death in those guts (data not shown).

Intestinal tissue homoeostasis is governed by an intricate balance between stem cell renewal, differentiation, and cell death coordinated by the action of several highly conserved molecular pathways [54]. For example, the WNT pathway maintains the renewal of ISCs, the Notch pathway contributes to cell fate decisions, and TGFβ pathway enforces differentiation and apoptosis to maintain the healthy turnover of intestinal epithelium. Perturbation in any of these pathways including deregulated TGFβ signalling contributes to developmental disorders and cancer [55, 56]. Mouse intestinal organoid model is a powerful tool to study the mammalian gut epithelial biology [57]. We did not find any overt TGFβ phenotypes in mouse intestinal organoids targeted by sg*Trip12*. However, when challenged by low dose TGFβ, sg*Trip12* organoids rapidly regressed to apoptotic looking rounded bodies with dark lumen suggestive of enhanced cell death. Indeed, in sg*Trip12* organoids TGFβ treatment sharply blocked ISCs proliferative index and dramatically enhanced apoptotic cell numbers compared to the wildtype controls. These results have broader implications since the precise functional biology of TRIP12 in the mammalian gut have not been tested before. Our data demonstrate that TRIP12 is an essential mediator of TGFβ signalling in the intestine and warrants further evaluation of TRIP12 function in intestinal biology and in colorectal cancer in vivo.

Finally, cancer cell metastasis and invasion are important steps during cancer dissemination and spread to distant organs. TGFβ signalling is a major contributor to these processes by favouring the process of EMT and by enhancing the gene expression of metastatic genes [58]. *TRIP12* inhibition increased the metastasis of MDA-MB-231 breast cancer cells compared to the controls. Importantly, these cells are highly metastatic and have previously been shown to possess mesenchymal properties [59]. Our finding that *TRIP12* inhibition further enhances the migratory properties of these cells suggest that TGFβ not only helps establish the process of EMT but also promotes metastasis of already established mesenchymal cells and TRIP12 is essential for regulating this phenomenon.

## Materials and methods

### Cell lines

Cell lines including HEK293T, HEK293FT, HCT116 and MDA231 were obtained from Cell Services at the Francis Crick Institute. The human Hepatocellular carcinoma cells (HepG2) & mouse immortalised NIH3T3 fibroblasts were a kind gift from Dr Johan Ericsson. HepG2 cells were maintained in minimal essential media (MEM) supplemented with 10% foetal bovine serum (FBS), non-essential amino acids (NEAA), sodium pyruvate, 1% Glutamax, 1% penicillin/streptomycin (Gibco), and 0.1% Normocin (Invivogen). All the other cell lines were maintained in DMEM High glucose (Gibco) supplemented with all the above reagents as for HepG2 cells except NEAA and sodium pyruvate. All cell lines were maintained in a standard cell culture humidified incubator at 37 °C and 5% CO2.

### Cloning and plasmids

The plasmids (pGEX-4T-1, Sigma-Aldrich) for recombinant glutathione S-transferase (GST), the GST-tagged SMAD proteins (SMAD 2 & 4), and Myc-SMAD4 (pcDNA3.0) were from Dr Johan Ericsson’s lab. GFP-tagged wildtype TRIP12, ΔHECT mutant, and ΔHECT/ΔWWE double mutant were reported before [23]. HA-Ubiquitin, Flag-TRIP12 wildtype and C1959A mutant are published before [23]. GFP-tagged TRIP12 ARM, IDR, ARM/IDR, and HECT domains were generated via Gateway cloning method (Invitrogen). Briefly, respective TRIP12 regions were PCR amplified using Flag-TRIP12 plasmid as a template with high fidelity Accuprime DNA polymerase (ThermoFisher). The PCR products were gel purified and 100 ng purified product and 150 ng pDONR221 was used for a BP reaction as given in Gateway cloning kit’s protocol. The insert/pDONR221 ligated mixture from the BP reaction was used to transform chemically competent DH5 alpha (NEB) *E coli* and at least 3 colonies were picked for colony PCR validation. At least one validated colony was further picked for plasmid miniprep DNA preparation using the kit’s protocol (GeneJet Plasmid Miniprep, ThermoFisher). The validated entry clones were then used for shuttling the TRIP12 regions to pcDNA6.2/N-EmGFP-DEST plasmid (V35520, Invitrogen) using the kit’s protocol. Separately, Gateway cloning was used to shuttle wildtype TRIP12 to pLenti CMV/TO GFP-Zeo DEST which as a gift from Eric Campeau & Paul Kaufman [60] (#17431, Addgene).

### Genetic targeting of mammalian cell lines

The CRISPR/Cas9 mediated *TRIP12* knockout of HEK293T and HCT116 cells were generated in a previous study [23]. MDA231 *TRIP12* knockout pools were generated exactly as before [23]. The stable HepG2 cells overexpressing a control or a *TRIP12* specific short hairpin (sh)-RNA were generated in this study. The cells were incubated with lentivirus supernatant containing either control or sh*TRIP12* (Sigma-Aldrich) for 24 h. After 48 h of transduction the media was changed to 1 μg/ml puromycin media and the cells were selected for another 48 h. The HEK293T *TRIP12* knockout pools stably overexpressing sh*SMAD4* (#37046, Addgene) were generated in this study. First, HEK293T cells were transiently co-transfected with plasmid overexpressing Cas9 and a cr*TRIP12* (Horizon) as before [23]. Second, lentivirus mediated stable expression of shS*MAD4* was done and cells selected in puromycin as above. The stable NIH3T3 fibroblasts overexpressing a control and two different sh*Trip12* (GIPZ, Horizon), were generated by incubating the NIH3T3 cells with the respective lentiviral supernatant as above. Stable cells were selected with 1 μg/ml puromycin (Sigma-Aldrich) for 48 h before propagation and experiments. The knockdown of *TRIP12* or *Trip12* was confirmed by Western blotting and by qRT-PCR respectively.

### Recombinant proteins’ purification

GST-tagged proteins were purified inhouse by overexpressing pGEX-4T-1 containing ORFs in codon optimised *E coli* (BL21 Codon + , Agilent). Briefly, GST-tagged ORFs were used to transform *E coli* and at least 3 distinct colonies were picked for each plasmid and grown overnight in 100 μg/ml ampicillin (Gibco) containing Luria-Bertani (LB) medium at 37 °C on a 200 rpm shaking platform. Bacterial cultures were incubated with isopropyl β-D-1-thiogalactopyranoside (IPTG) at 37 °C and after 2–4 h (h) of IPTG induction, a small aliquot of each colony culture was mixed with 4X SDS loading buffer and the extracts were boiled at 95 °C for 5 min, ran on 8% Tris-TGX gels, transferred to nitrocellulose membrane, blocked in 5% milk-TBST for 1 h at room temperature (RT) and blotted overnight with anti-rabbit GST sera (Invitrogen) to confirm expression of recombinant proteins. The colony culture giving the maximum yield of GST-tagged protein was further grown into 0.5 to 1 liter culture overnight and protein expression was induced next morning as given above. After 4 h of IPTG induction, bacteria were pelleted at 4000 rpm (Avanti J-265, Beckman Coulter) for 30 min in 4 °C, lysed in bacterial lysis buffer (ThermoFisher), sonicated periodically on ice, and ultracentrifuged at 22,000 rpm for 2 h (CP100NX, Hitachi). The cleared lysates were further incubated overnight in 4 °C with glutathione beads (Promega) for recombinant proteins enrichment. The next day, glutathione beads were washed 3 to 5X with 25 mM Tris containing wash buffer and eluted in 500 mM glutathione containing sample buffer (25 mM Tris 100 mM NaCl). Glutathione was removed by buffer exchange at 4 °C using appropriate molecular weight (MW) cut off columns (Thermofisher).

The construct for HECT domain of TRIP12 was amplified from the full length human TRIP12 ORF and subcloned into the pET28a-SUMO plasmid using the BamHI and XhoI restriction sites with N-terminal fusion with 6X HIS-SUMO tag. The resultant plasmid was transformed into *E coli* BL21 (DE3)-RIL competent cells. A single colony of *E coli* BL21(DE3)-RIL strain carrying pET28a-SUMO-HECT was inoculated in 100 ml of LB medium supplemented with 50 μg/ml kanamycin and 50 μg/ml of chloramphenicol. An aliquot (1%) of the overnight-grown culture was inoculated in fresh LB medium supplemented with the same antibiotics and left shaking at 200 rpm for 5 h at 37 °C. The cultures were then down tempered to 18 °C for 1 h before induction with 200 nM IPTG for 16 h at 18 °C. The cells were harvested by centrifuging the culture at 8000 rpm (Avanti J-265, Beckman Coulter) for 15 min at 4 °C. The cell pellet was resuspended in lysis buffer (50 mM Tris-HCl, pH 8.0, 500 mM NaCl, 10% glycerol, 5 mM β-mercaptoethanol (βME), and 20 mM Imidazole along with 0.2 ml of a mixture of protease inhibitors. Cell lysis was performed chemically, by incubation with hen egg white lysozyme, DNase I, and deoxycholate for about 1 h, incubated on ice. The soluble and insoluble cell fractions were separated by centrifuging the cell lysate at 18,000 rpm (CP100NX, Hitachi) for 60 min at 4 °C. The supernatant was loaded onto an immobilised metal affinity column (nickel-nitrilotriacetic acid column) that was pre-equilibrated with equilibration buffer (50 mM Tris-HCl, pH 8.0, 500 mM NaCl, 10% glycerol, 5 mM βME, and 20 mM Imidazole). The column was washed with 25 column volumes of wash buffer (50 mM Tris-HCl, pH 8.0, 500 mM NaCl, 10% glycerol, 5 mM βME, and 40 mM Imidazole) to get rid of any nonspecifically bound proteins. Finally, the bound HECT protein was eluted with elution buffer (50 mM Tris-HCl, pH 8.0, 500 mM NaCl, 5% glycerol, 5 mM βME, and 300 mM Imidazole) and subsequently dialysed overnight against 25 mM Tris-HCl, pH 8.0, 500 mM NaCl, 5% glycerol, 5 mM βME. The tag was removed by overnight digestion with Ulp protease during dialysis. To separate the cut and uncut fraction, the dialysed protein was again passed through the nickel-NTA column and the flow through was collected. The collected protein was concentrated and was further purified by gel filtration chromatography using a HiLoad 16/600 Superdex 200 column equilibrated with 25 mM Tris-HCl, pH 8.0, 300 mM NaCl, 5% glycerol, 3 mM DTT.

### Commercially sourced recombinant proteins

Human recombinant TGFβ1 (240-B-500/CF) was from R&D systems and TGFβ3 (RP8600) was from ThermoFisher. Recombinant His-UBE1 (E-305-025), His-UBCH5 (E2-616-100), His-UBCH7 (E2-640-100), and His-Ubiquitin (U-100H) were all from R&D Systems.

### Western blot antibodies

Anti-TRIP12 antibody was from Novus (NB100-97822). antibodies against anti-SMAD2 (51-1300), anti-phospho SMAD2 (44244 G), Anti-SMAD4 (PA5-95245) and anti-beta Actin (MA515739) were from Invitrogen. Anti-SMAD2/3 (5678), anti-Smurf2 (12024) and anti-SMAD4 antibodies were from (Cell Signalling Technologies). Anti-GFP (11814460001) antibody was from Roche. HRP-conjugated anti-mouse (SAB3701106) and anti-rabbit (65-6120) secondary antibodies were from Invitrogen.

### Immunoprecipitation (IP) and Mass Spectrometric (MS) Proteomics

Endogenous TRIP12 IP and MS proteomics were performed as before [61]. Sub confluent HCT116 cells were treated with a proteasome inhibitor (MG132) for 6 h. Cells were washed with ice-cold PBS and lysed in 1X cell lysis buffer (CLB; Cell Signalling Technology, #9803) or 1% Triton lysis buffer supplemented with protease inhibitors, PMSF (1 mM), and sodium fluoride (5 mM). Lysates were sonicated briefly and cleared by centrifugation for 5 min at 21,130 × g. Equal concentration and volume of cell lysates were pre-cleared with protein A/G Sepharose (Invitrogen) for 20 min at 4 °C. Precleared cell lysates were incubated with respective antibodies or control IgG (up to 5 μg/ml) on a rotating shaker in 4 °C overnight. Next morning, 30 μl of protein A/G Sepharose beads were added to each tube and further incubated for 3 h in 4 °C. Immuneprecipitates and beads complex were then washed three to five times in ice-cold lysis buffer supplemented with Halt^TM^ Protease Inhibitor Cocktail (ThermoFisher). Immunoprecipitated proteins were eluted in 4X SDS laemilli sample buffer. The eluted protein complexes were separated by SDS-PAGE on 8% Tris-HCl gel for 5–10 min at 150 volts. The gels were washed with distilled water and stained with instant blue Coomassie (Invitrogen) for 1–2 h and destained by three washes in distilled water. Separated and stained protein bands were excised by using a sterile razor blade placing the gel on top of a clean and clear piece of glass thoroughly cleaned with distilled water and 100% methanol. Each of the excised bands were placed into a sterile microcentrifuge tube (1.5 ml) and labelled carefully. The samples were subjected to MS analysis as previously described [61, 62]. The corresponding negative control lanes were also excised and analysed by MS, to ensure specificity in protein identification.

### Immunoprecipitations and Western blotting

Protein complexes were immunoprecipitated and resolved by SDS-PAGE as described above, transferred to nitrocellulose membrane (ThermoFisher), blocked in 5% BSA/TBST, and incubated with the indicated antibodies in 4 °C overnight. Next morning, membranes were washed in TBST for a total of three times, 15 min each and then incubated with HRP-conjugated anti-mouse (1:5000 dilution) or anti-rabbit (1:10.000 dilution) secondary antibodies in 5% non-fat dry milk/TBST for 1 h on a rotating end over shaker at RT. After secondary antibody incubation membranes were washed three times quickly with distilled water to remove residual milk-antibody solution, and then washed in TBST three times for 15 min each. After the final wash, immunoblots were visualised by an enhanced chemiluminescent (ECL) HRP substrate (SuperSignal^TM^ West Pico PLUS, ThermoFisher) on a chemiluminescence gel documentation equipment (Gel doc, Biorad). For denaturing ubiquitin pull-down assays, cell pellets were first denatured in TBS/1% SDS/5 mM DTT (TSD) buffer and boiled for 10 min at 95 °C. The denatured extracts were sonicated and spun at high speed for 10 min at 21,130 × g. The lysates were then resuspended in buffer TNN (50 mM Tris pH 7.5, 250 mM NaCl, 5 mM EDTA, 0.5% NP-40) to dilute the SDS concentration. 50% volume of each sample lysate was incubated in 4 °C overnight with either HA-conjugated Protein Sepharose A/G (ThermoFisher) to elute the total ubiquitinated proteins or with the Myc-conjugated Protein Sepharose A/G beads (ThermoFisher) to elute the target protein to confirm similar levels of expression throughout all the samples. Immune complexes were washed 3X in ice cold TNN buffer after overnight incubation and processed for Western blotting as described above.

### Luciferase reporter assay

The TGFβ responsive luciferase reporter assays were published before [63]. Briefly, cells were transiently transfected with 12XCAGA or 4XPAI luciferase synthetic promotors. All cells were transfected with GFP as a control to normalise the relative luciferase units. 24 h post-transfection, the cells were synchronised in 0.5% FBS media for 16 h. Next morning, the media was replaced with 0.5% FBS media ± 10 ng/ml TGFβ for 4 h. Cells were then washed and lyszed in luciferase reaction buffer and freeze thawed for efficient lysis of the monolayer. The lysates were then spun at 21,130 × g for 30 min to remove the cellular debris and luciferase signal was developed with the luciferase assay reagent kit (E1483, Promega) on a 96 well white background plate. Dual luciferase and GFP signals were recorded on a TECAN multiplate reader.

### GST-pull-down assay

Purified recombinant GST-SMAD2 and SMAD4 proteins were incubated with HEK293T wildtype cellular extracts overnight. The interacting proteins were isolated by incubating the extracts with glutathione beads for up to 4 h at 4 °C. The lysates were washed three times in RIPA lysis buffer supplemented with Halt^TM^ Protease Inhibitor Cocktail (ThermoFisher). These samples were separated on the SDS–PAGE gel, proteins were blotted on a nitrocellulose membrane and detected with anti-TRIP12 and an anti-GST serum as above.

### Cycloheximide chase protein stability assay

For protein stability assays, cells were grown as above to approximately 80% confluency and then incubated with the protein translation inhibitor cycloheximide (20 μg/ml) for indicated time points. Cells were washed three times with ice cold PBS, then harvested and lysed in RIPA lysis buffer supplemented with protease and phosphatase inhibitors. Samples were then processed for Western blotting as given above.

### Sub-cellular fractionation

To isolate cytoplasmic and nuclear fractions, cell pellets were washed with ice cold PBS twice and then were lysed in fractionation buffer containing 20 mM HEPES pH = 7.4, 10 mM KCl, 2 mM MgCl_2_, 1 mM EDTA, 1 mM PMSF, 0.5 mM NaF, 0.5 mM Na_3_VO_4_ supplemented with Halt^TM^ Protease Inhibitor Cocktail (ThermoFisher) by incubating on ice for 15 min. Cell suspension was passed through a 27-gauge needle until all cells are lysed and homogenised. The processed samples were further incubated on ice for 20 min to ensure complete cell lysis. Cell lysates were then separated by centrifugation at 21,130 × g for 15 min at 4 °C. The resultant supernatant was collected as ‘cytoplasmic’ fraction and stored at −80 °C. The remaining pellet was further processed for isolating the nuclear proteins by first resuspending in the fractionation buffer and then passing through a 25-gauge needle and centrifuging at 21,130 × g for 15 min at 4 °C. From this, the supernatant was discarded and the resulting pellet containing nuclei was resuspended in TBS supplemented with 0.1% SDS. The nuclear extracts were then sonicated and centrifuged at high speed to isolate the nuclear extracts from debris and insoluble fraction. Samples where then processed for Western blotting as given above.

### RNA extraction and quantitative Reverse Transcriptase (qRT)-PCR

RNA was extracted using GeneJet RNA Purification Kit (K0731, ThermoFisher). A total of 1000 ng RNA was used to synthesise cDNA using High-Capacity cDNA Reverse Transcription Kit (Applied Biosystems). For qRT-PCR, PowerUp SYBR Green Master Mix was used (Applied Biosystems). Primers used to amplify human TGFβ pathway genes were as follows *Actin*, forward (fwd): GGATGCAGAAGGAGATCACTG and reverse (rev): CGATCCACACGGAGTACTTG, *ADAM19* fwd: CAGCACTTGCCCCAAAGT and rev: CAGGTCTAGATTTTCGAGCTAATCA, *CTGF* fwd: CTCCTGCAGGCTAGAGAAGC, and rev: GATGCACTTTTTGCCCTTCTT, *PAI* fwd: AAGGCACCTCTGAGAACTTCA and rev: CCCAGGACTAGGCAGGTG, *P21* fwd: ATGTCAGAACCGGCTGGGGATG and rev: GGGCTTCCTCTTGGAGAAGATC, *SMAD7* fwd: GACAGCTCAATTCGGACAAC and rev: TCTCGTAGTCGAAAGCCTTG, and *TMEPAI* fwd: GCACAGTGTCAGGCAACGG and rev: AGATGGTGGGTGGCAGGTC.

### Cell migration assay

A total of 5 × 10^4^ MDA-MB-231 cells were suspended in 200 μl of serum-free DMEM medium and seeded into the upper chamber of each insert (24-well insert; pore size, 8 μm; BD Biosciences). Then, 700 μl of DMEM containing 10 ng/ml TGFβ were added to the lower chamber of a 24-well plate. After incubation at 37 °C for 12–16 h, migrated cells were washed (twice with PBS), and fixed with 4% paraformaldehyde (PFA). Fixed cells were permeabilized with 100% methanol and stained for 15 min in crystal violet solution (0.5% crystal violet in 25% methanol/PBS). Each insert was washed with PBS to remove excess stain. Cells that did not migrate to the lower compartment were removed with a cotton swab. Each insert was photographed in five random fields and total number of cells ± TGFβ treatment were counted by Fiji 2 software (Wayne Rasband, National Institute of Health, USA). The data is presented as fold change in number of migrated cells upon TGFβ induction.

### Lentivirus production and concentration

Lentivirus particles were produced in HEK293FT cells. The target and packaging plasmids were co-transfected (2nd or 3rd generation) using lipofectamine 3000 reagent. The cells were incubated with transfection mix overnight and the next morning the media was changed with fresh media. 48 h after transfection the media was collected and filtered through a 0.45 μm syringe filters and the viruses were stored in aliquots at −80 °C. Cells were transduced in regular media supplemented with polybrene (8 μg/ml). 24 h later, stable cells were selected in 1 μg/ml puromycin for up to 48 h. Stable pooled cells were propagated, validated, and use for experiments after validation.

Lentivirus particles were concentrated using either a homemade lentivirus concentrator (40% w/v PEG-8000, 1.2 M NaCl) or Lenti-X^ΤΜ^ concentrator (Takara Biosciences) using the supplier’s protocol. Briefly, 3 parts of sterile filtered lentiviral supernatant from HEK293FT cells was incubated with 1 part of lentivirus concentrator and incubated at 4 °C overnight on rotating platform. The next morning, precipitates were pelleted at 1600 × g in 4 °C for 60 min, supernatant discarded, and the pellets were resuspended either in organoids transduction media or in 1% sterile BSA/PBS solution.

### Zebrafish *trip12* model

Zebrafish (Danio rerio) wildtype (AB) adults were maintained in standard conditions under an approved protocol by the local Animal Care (QF-EVMC-IACUC 2020-1132). We used the CRISPR gene editing approach to knockout the zebrafish *trip12* ENSDARG00000061397. Two single guide RNA (sgRNA) targeting exon 1 sgRNA 1: GAATTAGGCCGGTTGGACAT, and exon 2 sgRNA2: AACAGCCTCTCTCTATCCGT were designed. The ribonucleoprotein (RNP) of the two sgRNAs and Cas9 mix was injected at 1-cell stage zebrafish embryos. At 24 h post-fertilisation, pooled embryos (*n* = 50) were collected for High Resolution Melting (HRM) curve assay. HRM was performed using Applied Biosystems in triplicates using 1 µl of the diluted sample in 20 µl reaction containing primers at final concentration of 300 nM, MeltDoc DNAs (AA, AG, GG) were run as controls and gave HRM profiles as expected in addition to the negative control. trip12 exon 1 primers: Fw TGAATGGTAGTGTCTAGAAAGGGT, Rev AGTGTGTGGTCTATTGGCTGGG with amplicon size 140 bp and exon 2 primers, fwd GGGTTCTCAAGTTCTTTCCTCTT, rev TGCTGCTTCCAAGTCTGGAT with amplicon size 100 bp.

### Zebrafish development characterisation

We examined the effect of CRISPR/Cas9 injection on zebrafish survival rate and gross morphology of the developing embryo. The development was assessed at 48 h post-fertilisation in comparison to the control. The phenotype classification was scored as G1: severely affected development, G2: mildly affected development, G3: Normal development.

### *Drosophila* lines

The following strains were obtained from the Bloomington *Drosophila* Stock Center: W1118 (#6326), UAS-*ctrip*-RNAi (#44481), UAS-*Medea*-RNAi (#33435). Dl-*Gal4*; Tub-Gal80ts, UAS-GFP flies were obtained from Lemaitre Bruno lab. For double knockdown we combined UAS-c*trip*-RNAi (II chromosome) with UAS-*Medea*-RNAi (III chromosome). Flies were reared on standard cornmeal/agar medium at 25 °C unless noted otherwise. The flies were transferred to fresh food vials every two days. Conditional expression in adult flies using tub-Gal80ts was achieved by maintaining flies at 18 °C until eclosion, then shifting young adults to 29 °C for 3 days before gut dissection.

### *Drosophila* gut Immunofluorescence

Experimental F1 flies were sorted, their external appendages were removed, and an intact gastrointestinal tract was dissected in 1X PBS and fixed in PBST (PBS with 0.1% Triton) containing 4% paraformaldehyde for 30 min. Three to six fly guts were processed as one experimental sample. Samples were then rinsed with PBST three times (15 min each) and blocked for 1 h in PBST containing 1% BSA. After blocking, samples were stained at 4 °C overnight with the mouse anti-Prospero (DSHB: MR1A, 1:20) primary antibody prepared in blocking buffer. Fly guts were washed with PBST three times and incubated with secondary antibodies (1:200) in blocking buffer at room temperature for 1 h in the dark. The secondary antibody used was goat anti-mouse conjugated to Alexa-568. Fly guts were then washed with PBST three times, mounted in Vectashield (Vector Laboratories) containing DAPI (2 µg/mL). Images were captured with a spinning disk confocal microscope (Yokogawa CSU-X1) equipped with 10x and 20x lens. All images were adjusted and processed using Fiji 2.

### Flow cytometric analysis

An intact gastrointestinal tract was dissected in 1 × 1% BSA/PBS buffer. Three fly guts were processed as one experimental sample. The isolated guts were subjected to enzymatic digestion by addition of 0.5% Trypsin-EDTA solution to each sample, vortexed and incubated at RT for 25–30 min. Digested guts were vortexed again and left to let the undigested midgut tissue sink to the bottom of the tube. The cells that were in suspension were collected in an eppendorf tube and gut digestion cycle was repeated on the undigested fraction until all the midgut tissue has been digested. Isolated cells were collected by centrifugation at 200 × g for 5 min at 4 °C, washed and stained with 50 µg/ml propidium iodide for live/dead cell separation. Dissociated cells from wildtype (e.g., w1118) *Drosophila* guts were used as controls. These cells were subjected to flow cytometry analysis and 10,000 cells were analysed per sample.

### Mouse intestinal organoid culture

Mouse intestinal culture was established as before [64]. Isolated intestines from C57BLK6 mice were placed in a 50 ml falcon tube containing 25 ml ice-cold PBS supplemented with 1% penicillin/streptomycin and vortexed several times. The washing cycle was repeated for a total of 4X or until the wash buffer appears transparent. Washed intestines were then cut open with sterile scissors or razor blade and chopped into approximately 1 cm pieces. The cut pieces were carefully washed, pelleted, cleared, and then incubated in 1 mM EDTA/PBS for 30 min at 4 °C. After this, the samples were shaken vigorously to dislodge the villi and the tissues were then allowed to settle, supernatant removed, and tissues were transferred to 5 mM EDTA/PBS for a further 1 h at 4 °C. After this, the samples were shaken vigorously, and the supernatant was collected and spun down at 300 × g for 5 min. The supernatant was then discarded, and the pellet was resuspended in 50 μl of Culturex Basement Membrane Extract (BME Path Clear, RnD systems) and seeded in a pre-warmed 48-well plate. The plate was incubated at 37 °C for 15 min to allow the BME to polymerise. Organoids were grown in mouse intestinal organoid media (Intesticult, Stem cells).

### Generation of CRISPR/Cas9 targeted intestinal organoids

3 to 5 days prior to lentivirus transduction, mouse intestinal organoids were trypsinized and plated in Intesticult media supplemented with 10 μm GSK3β inhibitor (CHIR, Tocris) 10 μm RHO kinase inhibitor (Y-27632, Tocris) and nicotinamide to obtain highly proliferating cystic organoids as established [65, 66]. On the day of transduction, at least 3 wells (100–200 organoids) were trypsinized and incubated with concentrated lentivirus (250 μl) in the media above supplemented with 8 μg/ml polybrene. The organoid-virus mixture was centrifuged at 600 × g for 1 h at 32 °C followed by incubation at 37 °C for at least 4 h to allow transduction. Transduced organoids were spun at 250 × g for 10 min, washed in ice cold Advanced DMEM F12 media (Gibco), and were resuspended and plated in BME on a 48-well plate. Stable organoids were selected in 1 μg/ml puromycin for 48 h after which, the selected pooled organoids were expanded and collected for validation of genetic manipulation via T7 restriction endonuclease assay.

### Validation of genetic targeting of *Trip12* locus

Genomic DNA was extracted from pelleted organoids cells using Lucigen QuickExtract™ DNA Extraction Solution (QE09050). A 426 bp product of mouse *Trip12* Exon 3 was amplified using 2 μl extracted DNA template by a forward (Fwd: GACAGGTTTACTCATGACAGGC) and a reverse primer (Rev: CACAAGAACTCTCTGTCCTC) in a PCR reaction using high fidelity AmpliTaq Gold DNA Polymerases (ThermoFisher). Amplified PCR products were column purified using GeneJET PCR purification kit (K0702, ThermoFisher) and then analysed on a nano drop. 200 ng of purified PCR product in NEB Buffer 2 (New England Biolabs, M0302L) was then heat denatured 95 °C for 5 min followed by 95 °C for 30 s with a ramp rate of 2°/s in a thermocycler (BioRad). Samples were then mixed to form heteroduplexes by cooling down to 85 °C for 5 min followed by 85 °C for 10 s with a ramp rate of 0.1°/s and then to 19 °C for 5 min on a thermocycler as above. From this, 10 µl is removed as uncut fraction and rest of the product is treated with 0.7 µl of T7 endonuclease enzyme (New England Bio Labs) at 37 °C for 20 min. The reactions were either kept at −20 °C or used immediately for gel electrophoresis.

### Immunofluorescence (IF) of organoids

Budding organoids were disintegrated in ice cold Advanced DMEM F12 media, spun at 250 × g for 5 min, and then plated as 20 μl mixture in BME on 8-well chamber slides (Sigma-Aldrich). 3 to 5 days after plating, the organoids were washed with 1X PBS and the cells were fixed in 4% paraformaldehyde (ThermoFisher) for 30 min at RT. The fixed organoids were washed 2X in wash buffer (1% BSA/PBS) and then incubated in permeabilization buffer (0.8% Triton in PBS) for 15 min at RT. Organoids were washed again in PBS and then blocked in blocking buffer (5% BSA/PBS) for 1 h at RT. For IF, organoids were incubated with cleaved caspase-3 (CC3, BD Biosciences) antibody overnight at 4 °C, washed 3X in wash buffer and incubated with Alexa Fluor™ 555 conjugated donkey anti-rabbit antibody for 1 h at RT. Stained organoids were washed 3X and counterstained with Prolong^ΤΜ^ Gold Antifade DAPI (P10144, Invitrogen) and mounted with a glass coverslip for imaging as above. For labelling of proliferating ISCs and transit amplifying cells (TA), organoids were pulsed with 10 μm 5-ethynyl-2’-deoxyuridine (EdU) for 4 h. Fixation, permeabilization, and staining was performed using the Click-iT™ EdU Cell Proliferation Alexa Fluor™ 594 kit (C10339, Invitrogen). Stained organoids were counter-stained with DAPI and mounted for imaging as above.

## Statistical calculations

Statistics were done by Student’s *t* test unless otherwise stated. Grouped analyses were performed by One-way or Two-way ANOVA.

### Supplementary information

Supplementary Figs. 1 to 5

Author checklist

## Data Availability

Raw files of proteomics datasets are uploaded in publicly available database Zenodo (DOI: 10.5281/zenodo.8343476).
