# Supplementary material for: Control of TGFβ signalling by ubiquitination independent function of E3 ubiquitin ligase TRIP12
Source: Cell Death Dis. 2023 Oct 20;14(10):692. doi: 10.1038/s41419-023-06215-y (PMC10589240; doi:10.1038/s41419-023-06215-y)
Supplement: Supplementary file 1 — Supplementary Figs. 1 to 5 [file 41419_2023_6215_MOESM1_ESM.pdf]

Control of TGFβ signalling by ubiquitination independent function of E3 ubiquitin ligase TRIP12

Kripa S. Keyan et al

Supplementary Material (Supp Figures 1 to 5)

Supplementary Fig 1

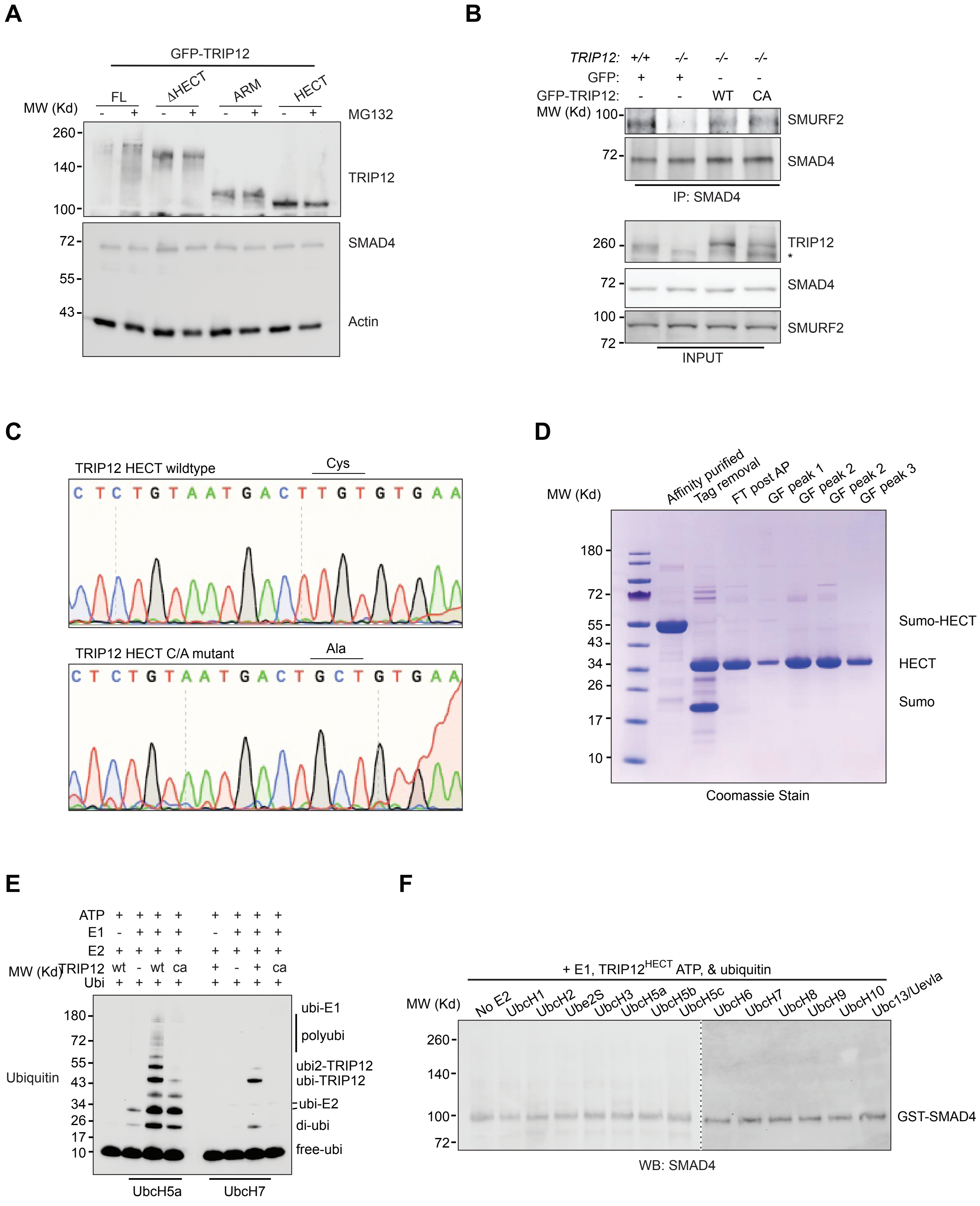

Supplementary Fig 2

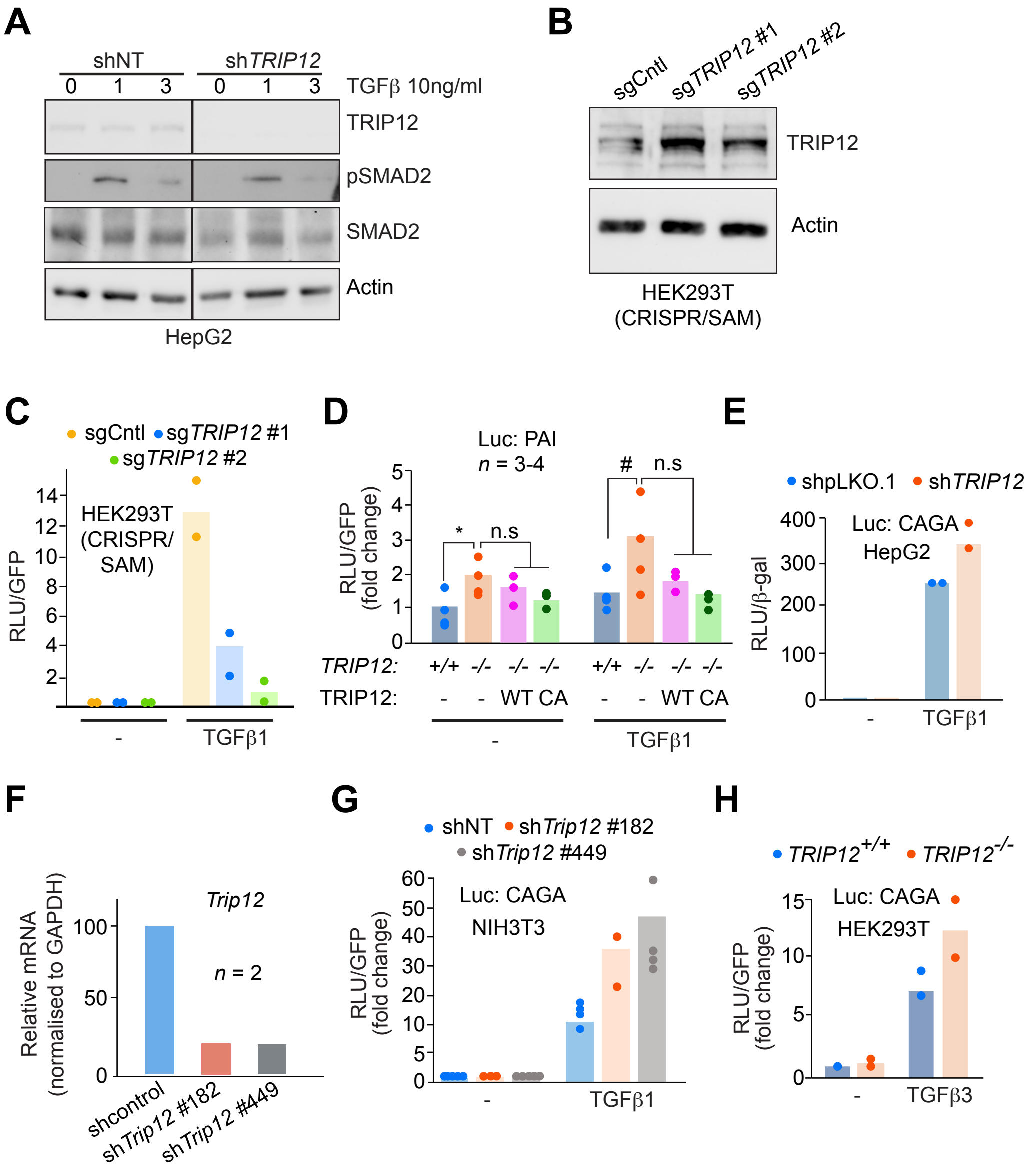

Supplementary Fig 3

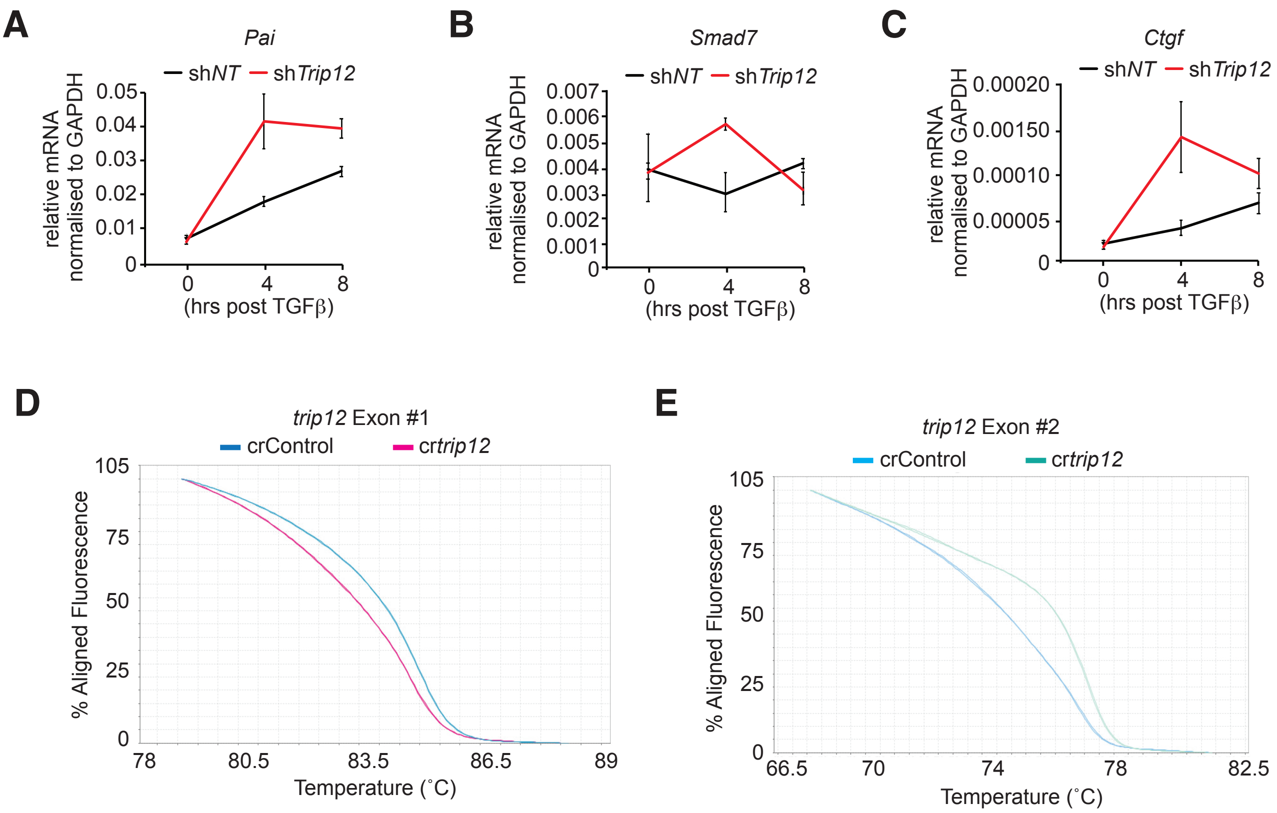

Supplementary Fig 4

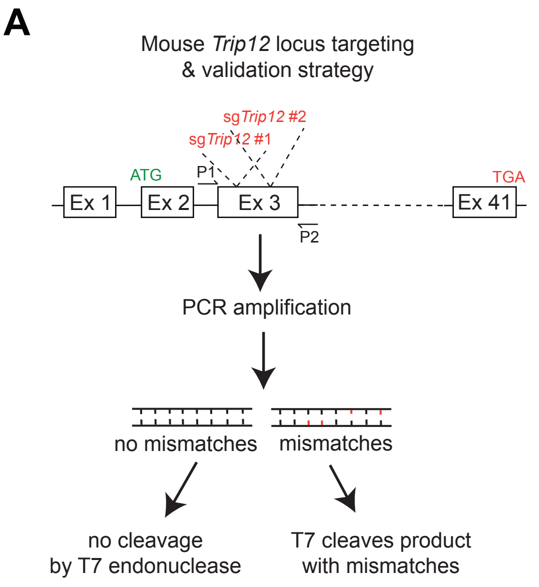

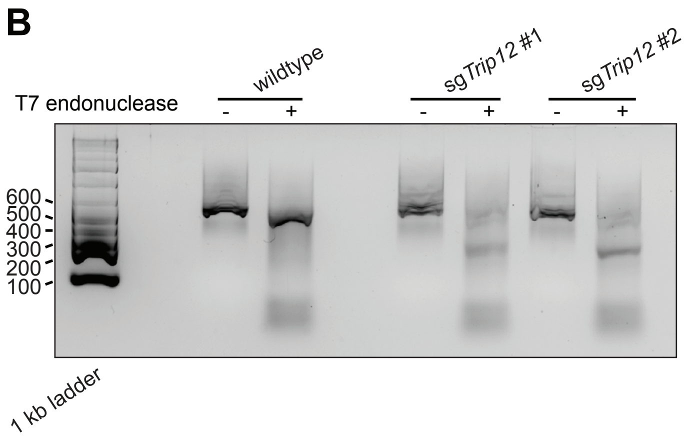

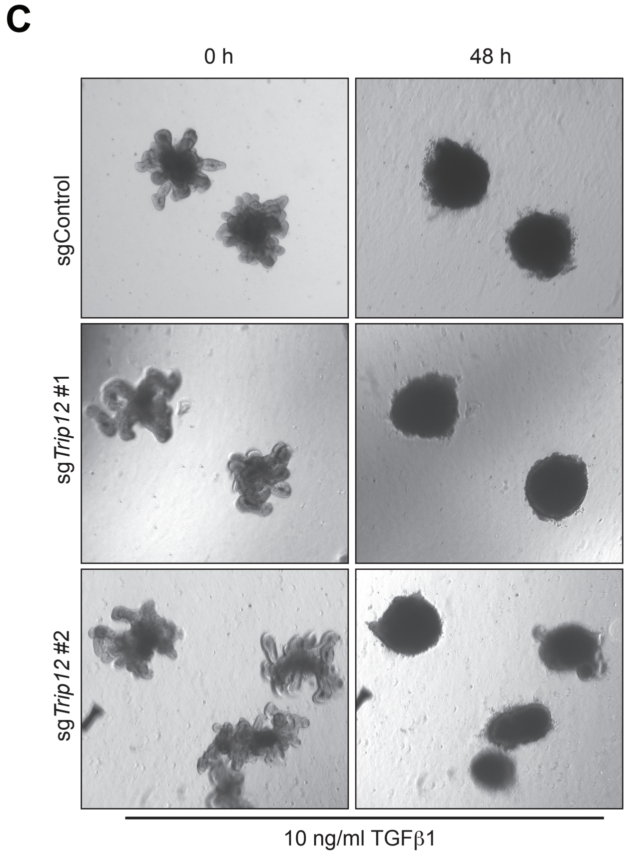

Supplementary Fig 5

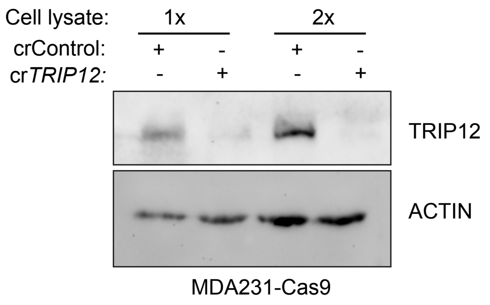

Supplementary Figure Legends

Supplementary Figure 1 (Related to Figure 2). A, Western blot showing endogenous SMAD4 levels in HEK293T cells overexpressing indicated constructs. **B,** SMAD4 IP followed by Western blots against indicated proteins. Lower panel shows Western blots against indicated proteins in 10 % input. **C,** Sanger sequencing validation of TRIP12 wildtype and C1959A construct. **D,** Coomassie confirmation of bacterial overexpression of TRIP12 HECT domain. **E,** *In vitro* ubiquitination assay in presence of indicated reagents. **F,** *In vitro* GST-SMAD4 ubiquitination assay in presence of indicated reagents.

Supplementary Figure 2 (Related to Figure 3). A, Western blot showing endogenous p-SMAD2 levels in HepG2 cells expressing indicated shRNAs. **B,** Western blot showing endogenous TRIP12 levels in HEK293T cells stably expressing CRISPR SAM sgcontrol (Cntl) or sg*TRIP12.* **C,** Luc:CAGA transcriptional reporter assay in HEK293T cells stably expressing CRISPR SAM sgcontrol or sg*TRIP12*. **D,** TGFβ Luc:PAI transcriptional reporter assay performed in presence or absence of indicated TRIP12 plasmids. **E,** Luc:CAGA transcriptional reporter assay in HepG2 cells expressing indicated shRNAs. **F,** qRT-PCR validation of *Trip12* knockdown expressing indicated shRNA in NIH3T3 cells. **G**, Luc:CAGA reporter assay in NIH3T3 cells expressing indicated shRNAs. **H,** Luc:CAGA transcriptional reporter assay in HEK293T cells with indicated genotypes in response to TGFβ3.

Supplementary Figure 3 (Related to Figure 4). A–C, qRT-PCR on indicated genes from NIH3T3 cells expressing a control and sh*Trip12*, bars are standard deviation. **D**, High-resolution melting (HRM) validation of *trip12* exon 1 genetic manipulation in zebrafish embryos **E,** High-resolution melting (HRM) validation of *trip12* exon 2 genetic manipulation in zebrafish embryos.

Supplementary Figure 4 (Related to Figure 7). A, CRISPR/Cas9 strategy for targeting mouse *Trip12* gene in intestinal organoids. **B**, T7 restriction endonuclease validation of *Trip12* locus manipulation in intestinal organoids expressing indicated sgRNAs. **C,** Bright field images of intestinal organoids with indicated genotypes and treated with 10 ng/ml TGFβ for 48 h.

Supplementary Figure 5 (Related to Figure 8). Western blot validation of *TRIP12* knockout in MDA-MB-231 cr*TRIP12* targeted cells.
